# Systematic Large Fragment Deletions in the Genome of *Synechococcus elongatus* and the Consequent Changes in Transcriptomic Profiles

**DOI:** 10.3390/genes14051091

**Published:** 2023-05-16

**Authors:** Feifei Hou, Zhufang Ke, Yi Xu, Yali Wang, Geqian Zhu, Hong Gao, Shuiling Ji, Xudong Xu

**Affiliations:** 1 College of Fisheries and Life Science, Dalian Ocean University, Dalian 116000, China; 2 Institute of Hydrobiology, Chinese Academy of Sciences, Wuhan 430072, China; 3 Hubei Key Laboratory of Genetic Regulation and Integrative Biology, School of Life Sciences, Central China Normal University, Wuhan 430079, China

**Keywords:** genome reduction, nonessential gene regions, transcriptomic profiles, growth conditions, CRISPR/Cpf1

## Abstract

Genome streamlining, as a natural process in the evolution of microbes, has become a common approach for generating ideal chassis cells for synthetic biology studies and industrial applications. However, systematic genome reduction remains a bottleneck in the generation of such chassis cells with cyanobacteria, due to very time-consuming genetic manipulations. *Synechococcus elongatus* PCC 7942, a unicellular cyanobacterium, is a candidate for systematic genome reduction, as its essential and nonessential genes have been experimentally identified. Here, we report that at least 20 of the 23 over 10 kb nonessential gene regions could be deleted and that stepwise deletions of these regions could be achieved. A septuple-deletion mutant (genome reduced by 3.8%) was generated, and the effects of genome reduction on the growth and genome-wide transcription were investigated. In the ancestral triple to sextuple mutants (*b*, *c*, *d*, *e1*), an increasingly large number of genes (up to 998) were upregulated relative to the wild type, while slightly fewer genes (831) were upregulated in the septuple mutant (*f*). In a different sextuple mutant (*e2*) derived from the quintuple mutant *d*, much fewer genes (232) were upregulated. Under the standard conditions in this study, the mutant *e2* showed a higher growth rate than the wild type, *e1* and *f*. Our results indicate that it is feasible to extensively reduce the genomes of cyanobacteria for generation of chassis cells and for experimental evolutionary studies.

## 1. Introduction

Genome reduction refers to the removal of dispensable regions from the genome, making microbial strains more suitable for serving as a chassis for downstream applications [1]. Benefits of genome reduction include creation of a cleaner genetic background, reduced consumption of energy and resources, and increased genome stability and yields of desired products, etc. [1,2,3]. In addition to applied research and potential industrial applications, genome reduction could also be employed to investigate the functions of certain genes or metabolic pathways, genome evolution, or even the origin of life [4].

Cyanobacteria are a group of prokaryotes that perform oxygenic photosynthesis and most often utilize sunlight as sole energy source and CO_2_ as sole C resource. Some cyanobacterial strains have been genetically engineered to produce biofuels or value-added products [5]. While genome reduction in other bacteria, such as *Escherichia coli* [6], *Bacillus subtilis* [7], *Streptomyces avermitilis* [8], and *Crynebacterium glutamicum* [9], has been extensively studied and while certain genome-streamlined strains showed better performance in the biosynthesis of target products, systematic genome reduction in cyanobacteria has not yet been reported. On the one hand, the essential and nonessential genes have not been fully identified in most cyanobacterial species; on the other hand, genetic manipulations in cyanobacteria are much more time consuming than those in other bacteria, and the multicopy chromosomes in cyanobacteria pose a challenge for obtaining fully segregated strains.

In recent years, CRISPR-based systems have been developed into a highly efficient technology for genome editing, including genomic deletions in cyanobacteria [10,11]. In CRISPR-Cas assisted genome editing systems, single-guide RNAs (sgRNAs) find the target sequences for the endonuclease Cas9 or Cas12a (formerly known as Cpf1) to cleave. Compared to Cas9, which appears to be toxic in a dose-dependent manner in certain species, Cpf1 is more suitable for genetic manipulations in cyanobacteria [11,12]. It has both a ribonuclease activity for processing the precrRNA array into mature crRNAs and a nuclease activity for cutting the double-stranded DNA sequence. The crRNA transcribed by the CRISPR array is responsible for guiding Cpf1 to the cutting sites [13,14]. The DNA breaks are repaired by the mechanism of homology directed repair. Employing the CRISPR/Cpf1 system and homologous sequences on the RSF1010-based broad-host-range plasmid, Ungerer and Pakrasi achieved gene insertion, knockout, and targeted mutation in *Synechocystis* sp. PCC 6803, *Synechococcus* sp. UTEX 2973, and *Anabaena* sp. PCC 7120 [11]. Using a derivative of the plasmid, Niu et al. showed that a single region up to 118 kb could be deleted in the genome of *Anabaena* sp. PCC 7120 [15]. As the plasmid that carries the CRISPR/Cpf1 system is replicative in the cyanobacteria, the target DNA sequences on the multi-copy chromosome are continually cleaved by gRNA-guided Cpf1, and the segregation of genetic modifications would be more efficient.

*Synechococcus elongatus* PCC 7942 (hereafter *Synechococcus* 7942), a unicellular cyanobacterium, is one of the model organisms for studies of photosynthesis [16,17,18] and circadian rhythms [19], because of its relatively fast growth, small genome, and simple genetic manipulations. It is also being developed as a microbial factory for the production of renewable fuels, chemicals, and pharmaceuticals [20,21,22,23]. In 2015, Rubin et al. reported the identification of 718 essential, 157 beneficial, and 1748 nonessential genes in 2723 genes of the whole genome [24]. This provided the basis for the prediction of large-fragment nonessential regions, and possibly for the generation of a minimal cyanobacterial cell.

In this study, we employed the CRISPR/Cpf1 system to systematically delete over 10 kb nonessential regions in *Synechococcus* 7942 and successfully reduced the genome size by 3.8%. To investigate the consequences of genome reduction, we followed the transcriptomic changes during stepwise deletions and compared the growth of genome-reduced strains with that of the wild type.

## 2. Materials and Methods

### 2.1. Strains and Culture Conditions

*Synechococcus* 7942 was provided by Prof. Xuefeng Lu at the Qingdao Institute of Bioenergy and Bioprocess Technology, Chinese Academy of Sciences. The wild-type *Synechococcus* 7942 and its derivatives (Table S1) were cultured in BG11 medium [25] at 30 °C in the light of 30 µE m^−2^ s^−1^. Antibiotics were supplemented to the medium as needed (spectinomycin at 25 g mL^−1^, kanamycin at 25 g mL^−1^).

The growth of *Synechococcus* strains was compared either in column photoreactors with aeration or in shaking flasks without aeration. For growth with aeration, cells cultured to the log phase were collected by centrifugation, washed with and resuspended in BG11, and the OD_730_ value was adjusted to 0.05.Then, 200 mL of cells were then grown in vertical column-type glass photoreactors (height: 48.5 cm; diameter: 2.0 cm) at 30 °C in the light of 50~150 µE m^−2^ s^−1^, bubbled with air or air supplemented with 1% CO_2_ at the aeration rate of 500 mL min^−1^. The OD_730_ was measured every 12 h. For growth without aeration, cells were cultured in 30 mL of BG11 in 50 mL flasks, starting from OD_730_ = 0.05, at 30 °C in the light of 30 µE m^−2^ s^−1^ on a rotary shaker (80 r min^−1^). The OD_730_ values were measured after 9 days.

### 2.2. Construction of CRISPR/Cpf1-Based Genome Editing Plasmids

The strategy was to replace the *lacZ* gene between the two Aar I sites on the pCpf1-Km or pCpf1-Sp plasmid with a synthetic double-stranded DNA bearing Aar I adapters on both sides, a PAM site and a gRNA sequence in the middle, followed by insertion of the homologous arms flanking the nonessential gene regions into the BamH I/Bgl II double digestion site. Briefly, two TTN sites were chosen as the PAM sites in each nonessential region, and the 22-bp sequences following these sites were used to generate the gRNA sequences. Aar I sites were added on both sides of the annealing oligos with PAM and gRNA sequences. The upstream and downstream homologous crossover arms were generated by fusion PCR using the primers listed in Table S2. Cloned DNA fragments were confirmed by sequencing.

Taking the genomic region 0051–0062 (for Synpcc7942_0051~Synpcc7942_0062) as an example, the oligos gRNA-0051-F1/R1 and gRNA-0051-F2/R2 were annealed (starting from denaturation at 95 °C for 1 min, then decreasing from 95 °C to 50 °C in 450 cycles, 0.1 °C per cycle for 8 s each, followed by incubation at 50 °C for 5 min and termination at 4 °C) to obtain the double-stranded oligonucleotides gRNA-0051-1 and gRNA-0051-2. The plasmid pCpf1-Km was digested with Aar I, ligated with gRNA-0051-1 or gRNA-0051-2, and transformed into *E*. *coli* DH5α. Transformants were identified by colony PCR using the primers AarI-F/R (Table S2), generating the plasmids pHB6582 and pHB6583 (Table S3).

Using the *Synechococcus* 7942 genome as the template, the upstream and downstream homologous arms of the 0051–0062 genomic region were obtained in the first step PCR using the primer pairs Cr-0051-F1/R1 and Cr-0051-F2/R2, followed by the second step (overlap) PCR to generate the fusion DNA for the 0051–0062 upstream and downstream sequences. The overlap PCR product was cloned into BamH I/Bgl II double digested plasmids pHB6582 and pHB6583 respectively, using a homologous recombination kit (Vazyme Biotech Co., Ltd. Economy & Technology Development Zone, Nanjing, Jiangsu, China), and the recombination products were transformed into *E*. *coli* DH10B, generating the plasmids pHB6588 and pHB6589 (Table S3). All editing plasmids were constructed likewise, and the plasmids are listed in Table S3.

### 2.3. Generation of Genome Deletions in Synechococcus 7942

Plasmids were introduced into *Synechococcus* 7942 and mutant strains by conjugation with *Escherichia coli* [26]. The exconjugants with genome deletion were screened with methods described previously [11,15]. The complete segregation of mutants was confirmed by PCR. *Synechococcus* 7942 strains are listed in Table S1.

### 2.4. RNA Extraction

Wild-type *Synechococcus* 7942 and its derivatives were cultured in 150 mL of BG11 in 250 mL flasks on a rotary shaker (80 r m^−1^) at 30 °C in the light of 30 µE m^−2^ s^−1^, with three biological replications. Cells were harvested by centrifugation when the OD_730_ was around 0.9. Total RNA was extracted from the cells using TRIzol^®^ Reagent according to the manufacturer’s instructions (Invitrogen, Waltham, MA, USA), and the residual genomic DNA was removed using DNase I (TaKaRa, Tokyo, Japan).

### 2.5. Library Preparation and Illumina Hiseq Sequencing

A total amount of 5 μg RNA per sample was used as the input material for RNA sample preparations. First, ribosomal RNA was removed with an Epicentre Ribo-zero™ rRNA Removal Kit (Epicentre, Madison, WI, USA), and the rRNA-depleted RNA sample was cleaned with ethanol precipitation. Subsequently, sequencing libraries were generated using the cleaned RNA sample with a NEBNext^®^ Ultra™ Directional RNA Library Prep Kit for Illumina^®^ (NEB, Ipswich, MA, USA), according to the manufacturer’s instructions. The clustering of the index-coded samples was performed on a cBot Cluster Generation System using a TruSeq PE Cluster Kit v3-cBot-HS (Illumia, San Diego, CA, USA). Afterwards, the libraries were sequenced by Illumina NovaSeq 6000 sequencing (150 bp*2, Shanghai BIOZERON Co., Ltd., Shanghai, China). The raw paired end reads were trimmed and quality controlled using Trimmomatic (version 0.36 http://www.usadellab.org/cms/uploads/supplementary/Trimmomatic, accessed on 10 August 2021). Then clean reads were separately aligned to the reference genome with orientation mode using hisat2 (https://ccb.jhu.edu/software/hisat2/index.shtml, accessed on 10 August 2021) software.

### 2.6. Differential Expression Analysis and Functional Enrichment

To identify DEGs (differential expression genes) between two different samples, the expression level of each gene was calculated using the fragments per kilobase of exon per million mapped reads (FPKM) method. Cuffdiff (http://cufflinks.cbcb.umd.edu/, accessed on 2 September 2021) was used for differential expression analysis based on Fisher’s exact test. The DEGs between two samples were selected based on a fold change greater than 2 and an FDR-adjusted *p*-value less than 0.05 following Benjamini–Hochberg correction for multiple testing. GO functional enrichment and KEGG pathway analysis were carried out using Goatools (https://github.com/tanghaibao/Goatools, accessed on 3 September 2021) and KOBAS (http://kobas.cbi.pku.edu.cn/ko-bas3, accessed on 3 September 2021) to annotate the functions of genes. DEGs were considered significantly enriched in GO terms and metabolic pathways when their Bonferroni-corrected *p*-value was less than 0.05.

### 2.7. Graphic Plotting of RNA-Seq

Volcano plotting was performed using OriginPro 2023 software (10.0.0.154 educational version, OriginLab Corporation, Northampton, MA, USA), and heat maps were plotted using the pheatmap package (https://cran.r-project.org/web/packages/pheatmap/index.html, accessed on 8 October 2022) in R.

## 3. Results

### 3.1. Identification of over 10 kb Dispensable Chromosomal Regions in Synechococcus 7942

**Figure 1:**
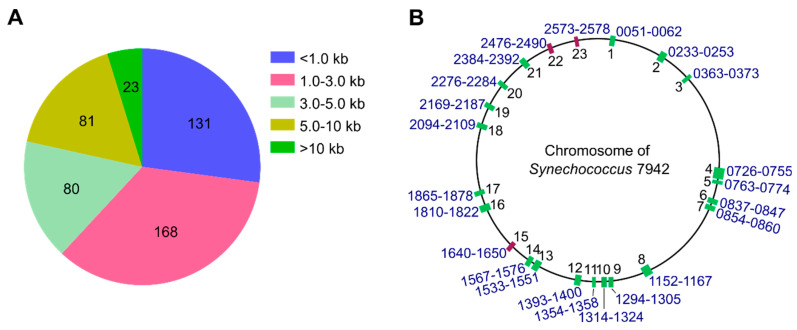
Nonessential genes in the genome of *Synechococcus* 7942. (**A**) The number (pie chart) and lengths (square blocks) of nonessential gene regions. The total number of nonessential genes is 1748 [24], forming 483 regions. (**B**) Locations of the 23 over 10 kb nonessential gene regions on the genome. The green boxes indicate the regions where deletions were completely segregated, whereas the red boxes indicate the regions where deletions could not be segregated.

In *Synechococcus* 7942, 1748 genes were identified as nonessential [24]. As the first step to construct a genome-streamlined strain of the cyanobacterium, we planned to delete these over 10 kb of nonessential gene regions. There are 23 such regions in the 2.75 Mb chromosome (Figure 1, Table S4), with a total size of 334,210 bp, accounting for 12.15% of the chromosome.

We employed the CRISPR/Cpf1 system [11,15] to delete the 23 chromosomal regions. Two plasmids, pCpf1b-Sp (spectinomycin resistance) and pCpf1b-Km (kanamycin resistance) [15], were used; for each target region, two gRNA sequences were designed (Table S2). Sixty nine genome-editing plasmids were constructed, as described in Table S3.

**Figure 2:**
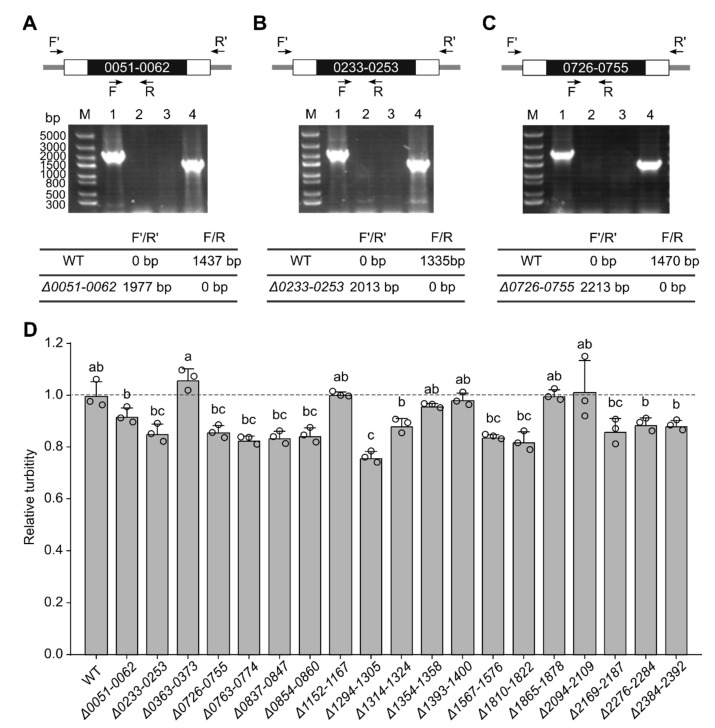
The PCR verification of single-deletion mutants and the growth of single mutants in shaking flasks. (**A**–**C**), PCR examinations of three representative single mutants, 0051–0062, 0233–0253, and 0726–0755. Results for other single mutants are shown in Figures S1 and S2. For each panel of (**A**–**C**), the upper part displays the relative positions of the oligonucleotides for PCR. The black boxes represent the deleted chromosomal regions, and the empty boxes represent the homologous recombination regions (the lengths are not proportional to the actual sizes). F, R, F′, and R′ are short names for the oligonucleotides Cr-0051-F, Cr-0051-R, Cr-0051-F′, Cr-0051-R′ (**A**), Cr-0233-F, Cr-0233-R, Cr-0233-F′, Cr-0233-R′ (**B**), and Cr-0726-F, Cr-0726-R, Cr-0726-F′, Cr-0726-R′ (**C**), respectively. The electrophoretograms in the middle show the PCR results. The sizes of DNA markers (M) are indicated on the left in (**A**). The templates for lanes 1 and 3 are the genomic DNA of single mutants, and for lanes 2 and 4 are the genomic DNA of WT (wild type). The primers for lanes 1 and 2 are F′/R′ and for lanes 3 and 4 are F/R. The expected sizes of the PCR products for WT, *Δ0051-0062* (**A**), *Δ0233-0253* (**B**), and *Δ0726-0755* (**C**) are shown at the bottom. (**D**) Relative turbidities of strains grown in shaking flasks. The OD_730_ values were measured after a 9-day cultivation in shaking flasks at 30 °C in the light of 30 µE m^−2^ s^−1^, and the relative turbidities were calculated taking the average of WT values as 1. The data are plotted as means ± s.d. (n = 3 independent biological repeats). The small open circles represent the individual data points. Statistically significant differences are indicated by distinct letters above the histograms. Groups that share the same letter indicate no significant differences, except for ab and bc, which are also significantly different as determined by one-way analysis of variance (ANOVA) and Tukey’s test (*p* < 0.05).

The editing plasmids were conjugatively transferred into *Synechococcus* 7942, and the exconjugants were examined with PCR. Taking the deletion regions 0051–0062 (for Synpcc7942_0051~Synpcc7942_0062, all other deletion regions are named likewise), 0233–0253 and 0726–0755 as examples (Figure 2A,C), the PCR products generated using primers F′/R′ (for primer names suffixed with -F′ and -R′) were only found in mutants, whereas the PCR products using primers F/R (for those with -F and -R) were only found in the wild type. Using the pCpf1b-Km-derived plasmids, we obtained 19 completely segregated single-deletion mutants (Figure S1, Table S1). Similarly, the pCpf1b-Sp-derived plasmids produced 17 (Figure S2), but one of them was an addition to the 19 deletion mutants (Figure S2, region 1533–1551). Therefore, at least 20 over 10 kb regions were shown to be ‘nonessential’.

The growth of the single-deletion mutants was compared with that of the wild type in shaking flasks. Under this condition, most deletions appeared to slow down the growth of *Synechococcus* 7942 (Figure 2D).

### 3.2. Systematic Large Fragment Deletions

**Figure 3:**
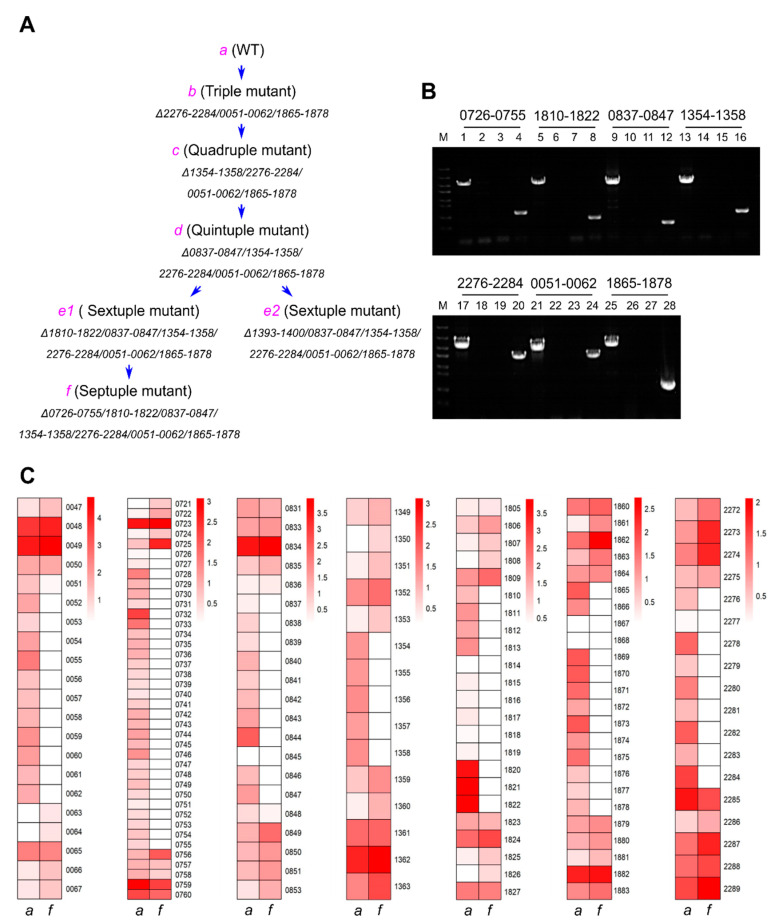
Genotype and transcriptomic analysis of the septuple mutant strain. (**A**) The flow chart for the generation of the septuple mutant, with the first and second deletion steps omitted. The deleted nonessential gene regions are indicated as abbreviated ORF numbers linked by a short dash (For example, 1354–1358 for Synpcc7942_1354~Synpcc7942_1358). (**B**) PCR verification on the genotype of the septuple mutant. DNA markers (M) are the same as those in Figure 2. The short dash-linked ORF numbers at the top stand for the deleted regions. The PCR templates for lanes 1, 3, 5, 7, 9, 11, 13, 15, 17, 19, 21, 23, 25, and 27 are the genomic DNA of the septuple mutant, and for lanes 2, 4, 6, 8, 10, 12, 14, 16, 18, 20, 22, 24, 26, and 28 are the genomic DNA of the WT. The PCR primers are Cr-0726-F′/R′ (lanes 1 and 2), Cr-0726-F-1/R-1 (lanes 3 and 4), Cr-1810-F′/R′ (lanes 5 and 6), Cr-1810-F-1/R-1 (lanes 7 and 8), Cr-0837-F′/R′ (lanes 9 and 10), Cr-0837-F-3/R-3 (lanes 11 and 12), Cr-1354-F′/R′ (lanes 13 and 14), Cr-1354-F/R-1 (lanes 15 and 16), Cr-2276-F′/R′ (lanes 17 and 18), Cr-2276-F/R (lanes 19 and 20), Cr-0051-F′/R′ (lanes 21 and 22), Cr-0051-F/R (lanes 23 and 24), Cr-1865-F′/R′ (lanes 25 and 26), and Cr-1865-F/-R-2 (lanes 27 and 28). (**C**) Heat map diagrams of expression levels of genes in the deletion and flanking regions in the septuple mutant (the *f* strain) and the wild type (the *a* strain), based on the averaged log_10_(FPKM+1) values of each gene. The color scale of white (low)/red (high) represents the expression levels of genes. Relative mRNA levels were determined using RNA-seq.

The 20 over 10 kb nonessential regions could be deleted sequentially by alternately using the two sets of Km- and Sp-resistance editing plasmids. As an example, Figure 3 shows how a septuple mutant (*f*) was generated step by step and examined. The mutant *f* was generated via the triple mutant *b*, the quadruple mutant *c*, the quintuple mutant *d,* and the sextuple mutant *e1*. From *d*, also derived another sextuple mutant *e2*. Figure 3A is the flow chart for the generation of the septuple mutant, Figure 3B shows the PCR examination result of the mutant. All seven regions were deleted, and the mutations were fully segregated. PCR examinations of those intermediate strains and the mutant *e2* are shown in the supplemental Figure S3. In addition, the complete segregation of the septuple mutant was confirmed by transcriptomic analysis based on RNA-Seq. In the seven deleted regions, there was no or very few RNA-seq reads mapped, in contrast to the corresponding regions in the wild type and the flanking regions in the mutant (Figure 3C; Table S5).

### 3.3. Large-Scale Gene Upregulation in Response to Genome Reduction

**Figure 4:**
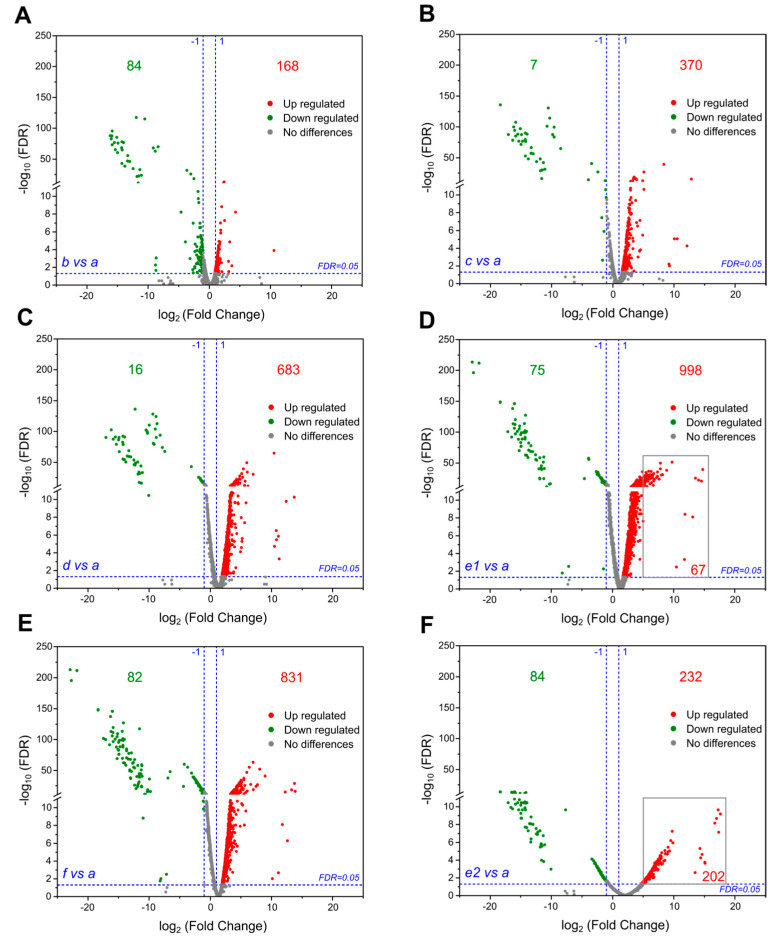
RNA-seq analysis of the strains obtained during the sequential deletions. (**A**–**F**) Volcano plots reporting −log_10_ (FDR) (*y*-axis) against log_2_ (fold change of gene expression) (*x*-axis) in the triple mutant (the strain *b*), the quadruple mutant (the strain *c*), the quintuple mutant (the strain *d*), the sextuple mutant (the strain *e1*), the septuple mutant (the strain *f*), and another different sextuple mutant (the strain *e2*), relative to the wild-type strain *a*. The numbers of upregulated (red) and downregulated genes (green, excluding the deleted genes) are indicated in each plot (FDR < 0.05). FDR: false discovery rate is the adjusted *p*-value after Benjamini–Hochberg correction for multiple testing.

To assess how the deletions of nonessential gene regions impacted the transcriptomic profiles of mutants, we analyzed the RNA-seq data of triple to septuple mutants and the wild type. As shown in Figure 4, genome deletions caused large-scale gene upregulation relative to the wild type. Except for the sextuple mutant *e2*, which displayed much fewer upregulated genes (Figure 4F), the number of such upregulated genes increased with the accumulation of deletions, peaking at 998 in the sextuple mutant *e1* (Figure 4A–D) and decreasing slightly to 831 in the septuple mutant *f* (Figure 4E). In contrast, the number of downregulated genes fluctuated between 7 and 84 (Figure 4A–E). While only 232 genes were upregulated in *e2*, 202 of them were upregulated more than 32-fold (Figure 4F), compared to 67 of 998 in *e1* (Figure 4D). However, the average FPKM value (for evaluation of the relative mRNA level, see Materials and Methods) of the 232 genes increased from 1.16 (WT) to 71.79 (*e2*), whereas that of the 998 genes increased from 20.13 (WT) to 131.15 (*e1*) (calculated based on Table S5). Therefore, the increased RNA synthesis in *e2* was actually far below that in *e1*.

### 3.4. Growth of Genome-Reduced Strains under Different Light and CO_2_ Conditions

**Figure 5:**
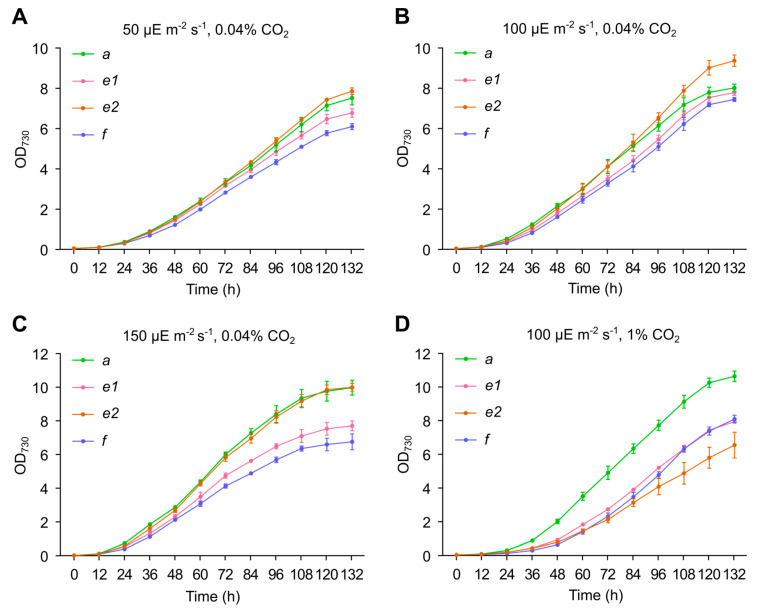
Growth curves of the wild-type (*a*), the sextuple mutants (*e1* and *e2*), and the septuple mutant (*f*) under different conditions. Cells were grown in column photoreactors at 30 °C, in light intensities of 50 µE m^−2^ s^−1^ (**A**), 100 µE m^−2^ s^−1^ (**B**,**D**), 150 µE m^−2^ s^−1^ (**C**), bubbled with air (~0.04% CO_2_) (**A**–**C**) or air supplemented with 1% CO_2_ (**D**). The initial OD_730_ was adjusted to 0.05 and grown at the indicated light intensities or CO_2_ concentrations, and OD_730_ was monitored every 12 h.

Nonessential genes may play an important role in different environmental acclimations [27]; therefore, genome deletions may affect the growth of cells under certain conditions; on the other hand, whether the large-scale gene upregulation (more consumption of resources in RNA synthesis) affects the growth rate remains to be answered. We compared the growth of the sextuple mutants *e1* and *e2*, the septuple mutant *f,* and the wild-type strain in column photoreactors at different light intensities and CO_2_ concentrations. Under the standard conditions (30 °C, 100 µE m^−2^ s^−1^, bubbling with air) in this study, mutants *e1* and *f* showed very slight differences from the wild type in growth (Figure 5B); *e2*; however, it showed an increased growth rate compared to the wild type, particularly at the late logarithmic phase. When the light intensity was decreased to 50 µE m^−2^ s^−1^ or increased to 150 µE m^−2^ s^−1^, *e2* and the wild type became similar to each other, whereas *e1* and *f* showed significantly slower growth (Figure 5A,C). When the CO_2_ concentration was increased to 1%, all the mutants showed slower growth than the wild type (Figure 5D). The increased light/CO_2_ availability apparently boosted the growth of the wild type but not the genome-reduced mutants (compare Figure 5B with Figure 5C,D); and the growth of *e2* was remarkably slowed down by the higher CO_2_ concentration (Figure 5B,D).

## 4. Discussion

The size of bacterial genomes can be greatly reduced through the removal of large fragments. However, deletions over certain ranges may cause lower growth rates [28,29], weaker environmental adaptability, poorer genetic stability [29,30], and even abnormal cell morphology [29]. Hitherto, the smallest size of a reduced bacterial genome (rather than synthetic genome) was 2.68 Mb [1,31], which is comparable in size to the *Synechococcus* chromosome (2.75 Mb). Although 1748 nonessential genes have been identified in *Synechococcus* 7942 [24], it is still unclear how many of them can be deleted stepwise. In this study, we showed that 20 of the 23 over 10 kb nonessential gene regions can be individually deleted and that multiple deletions resulted in a remarkable reduction in the size of the chromosome, as seen in the septuple mutant *f*. Now, we are on the way to deleting each of the 20 nonessential regions sequentially. Of the 23 predicted nonessential regions, the other 3 were not successfully deleted by any of the three sets of editing plasmids. This was most likely due to the presence of individually nonessential genes that could not be deleted simultaneously.

**Figure 6:**
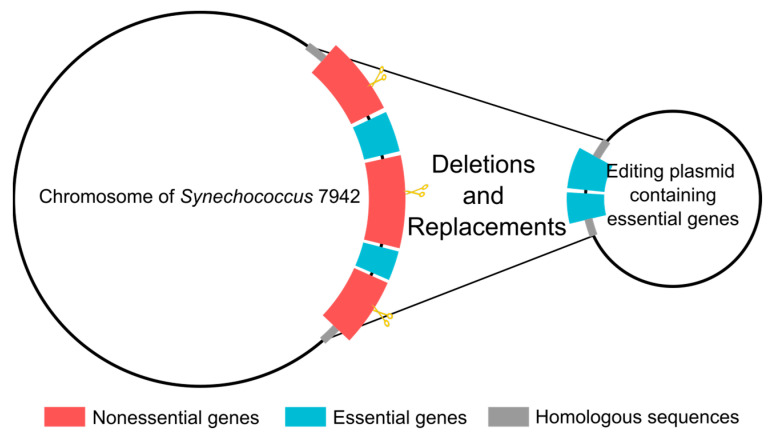
Proposed deletion-replacement strategy for removal of nonessential gene regions interrupted by essential genes. Deletions of such regions are accompanied by the reintroduction of the innocently deleted essential genes located in them. The scissors indicate the cutting sites in the genome.

In the genome, there are also many smaller nonessential regions that are separated by two or three essential genes. To delete these discontinuous regions, we can use a strategy that combines deletions and replacements. In particular, the separate essential genes can be assembled into one fragment between the two homologous arms in the editing plasmids, so that these essential genes are retained when the discontinuous nonessential gene regions are deleted as a whole (Figure 6).

To determine the effects of genome deletions on gene expression, we compared the transcriptomic profiles of the multiple deletion mutants with that of the wild type. However, we did not observe any pathways or functional categories of genes that were specifically responsive to the cumulative deletions. Nevertheless, we observed a stepwise increase in the number of upregulated genes relative to the wild type, with a slight decrease in the septuple mutant (Figure 4). One possible explanation for the large-scale gene upregulation could be the redirection of resources saved in DNA synthesis (due to genome reduction) towards RNA synthesis. However, in a different sextuple mutant (*e2*), there were much fewer genes upregulated and a significantly smaller increase in RNA synthesis (according to the evaluation with FPKM values). Therefore, large-scale gene upregulation is better explained as transcriptional responses to specific combinations of genomic deletions.

Genome deletions also affected the growth of *Synechococcus* 7942 under various conditions. In shaking flasks without aeration, most single-deletion mutants grew more slowly than the wild type. However, under standard aeration conditions, the sextuple- and septuple-deletion mutants exhibited similar or improved growth compared to the wild type. The increased growth rate of the sextuple mutant *e2* could be taken as an example of the role of genomic deletions in adaptive evolution in certain environments. We found that large fragment deletions decreased the growth rate of *Synechococcus* 7942 in most cases. According to a systematic study in *Escherichia coli*, there is a correlation between genome reduction and decreases in growth and cell density [3,28]. This appears to be a disadvantage for the genome reduction project. However, either wild type or genomically modified bacterial strains can be improved for better growth in laboratory adaptive evolution [3,32,33]. This strategy could also be practical for fitness recovery of the genome-streamlined strains of *Synechococcus* 7942.

## Data Availability

The data supporting the findings of this study are available within the article and its Supplementary Information.

## Supplementary Materials

The following supporting information can be downloaded at: https://www.mdpi.com/article/10.3390/genes14051091/s1, Figure S1: PCR verification of 19 single-deletion mutants; Figure S2: PCR verification of 17 single-deletion mutants generated by another sets of editing plasmids based on CRISPR/Cpf1b-Sp; Figure S3: PCR verification of multiple-deletion mutants; Table S1: List of wild-type and mutant strains; Table S2: Primers used in this study; Table S3: List of 69 editing plasmids in this study; Table S4: List of nonessential gene regions over 10 kb; Table S5: RNA-seq analyses. The RNA-seq raw data has been submitted to the NCBI Sequence Read Archive (SRA; http://www.ncbi.nlm.nih.gov/sra/ accessed on 3 February 2023) under accession number SRR23434692-SRR23434698.
