# Supplementary material for: Systematic Large Fragment Deletions in the Genome of Synechococcus elongatus and the Consequent Changes in Transcriptomic Profiles
Source: Genes (Basel). 2023 May 16;14(5):1091. doi: 10.3390/genes14051091 (PMC10217888; doi:10.3390/genes14051091)
Supplement: Supplementary file 1 [file genes-14-01091-s001.zip › Supporting information.pdf]

Systematic Large Fragment Deletions in the Genome of *Synechococcus elongatus* and the Consequent Changes in Transcriptomic Profiles

Feifei Hou [1,2,†], Zhufang Ke [2,†], Yi Xu [2], Yali Wang [3], Geqian Zhu [2], Hong Gao [2], Shuiling Ji [3,*] and Xudong Xu [2,*]

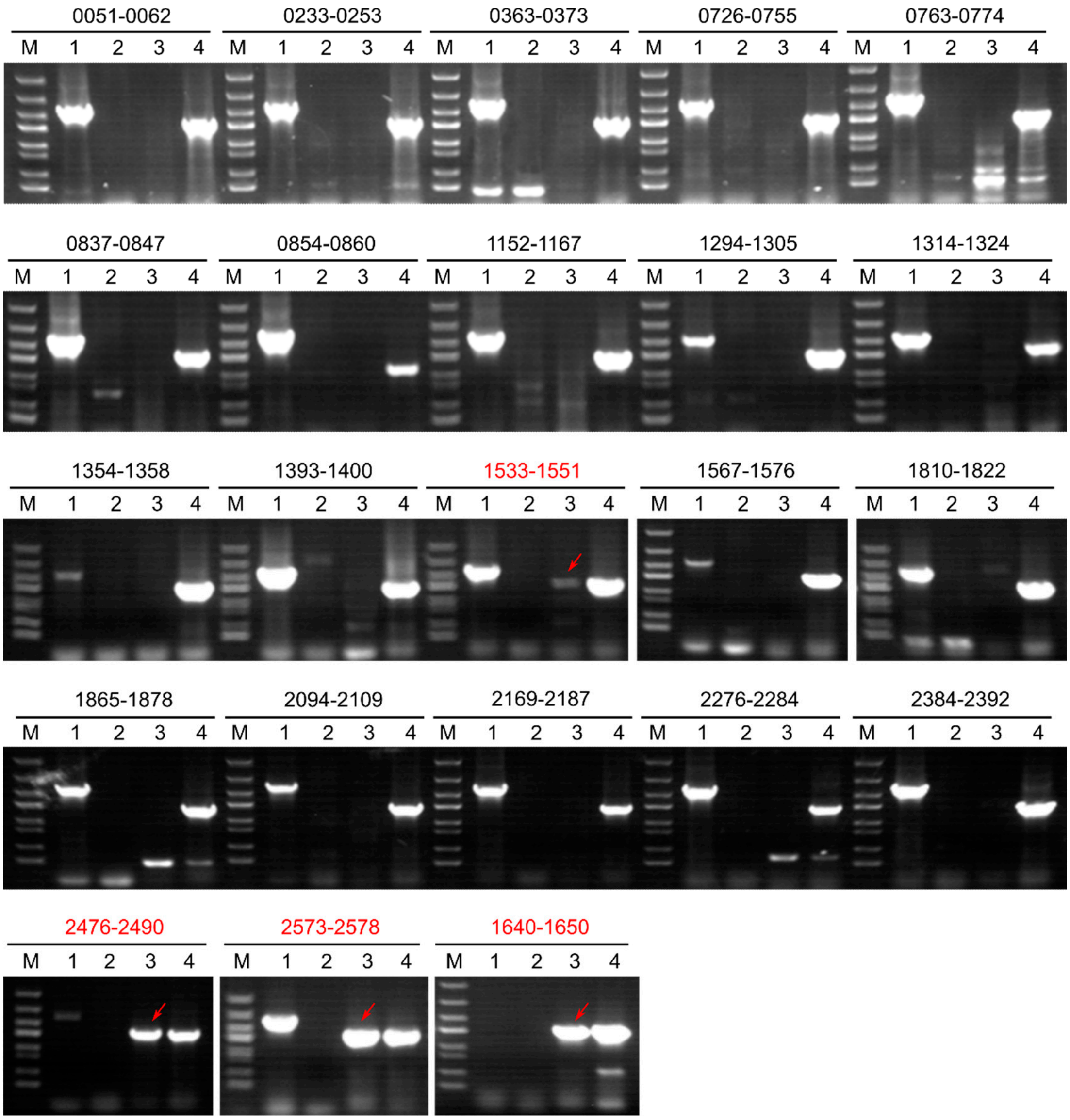

Figure S1. PCR examinations of the single-deletion mutants generated by using the editing plasmids derived from Cpf1b-Km. Nineteen nonessential-gene regions were completely deleted (numbers in black), while the other 4 regions were not (numbers in red). The red arrows indicate that the nonessential-gene regions were retained in the exconjugants as in the wild type. DNA markers (M) are the same as those in Figure S1. The short dash-linked numbers on the top stand for the deleted regions, indicated by the first and the last ORFs (For example, 0051-0062 for Synpcc7942_0051 ~ Synpcc7942_0062). The templates for lanes 1 and 3 are the genomic DNA of single mutants, and for lanes 2 and 4 are the genomic DNA of WT. The primers for lanes 1 and 2 are Cr-first ORFs-F′/R′ (For example, primers Cr-1865-F′/R′ for region 1865-1878), and for lanes 3 and 4 are Cr-first ORFs-F/R (For example, primers Cr-1865-F/R for region 1865-1878).

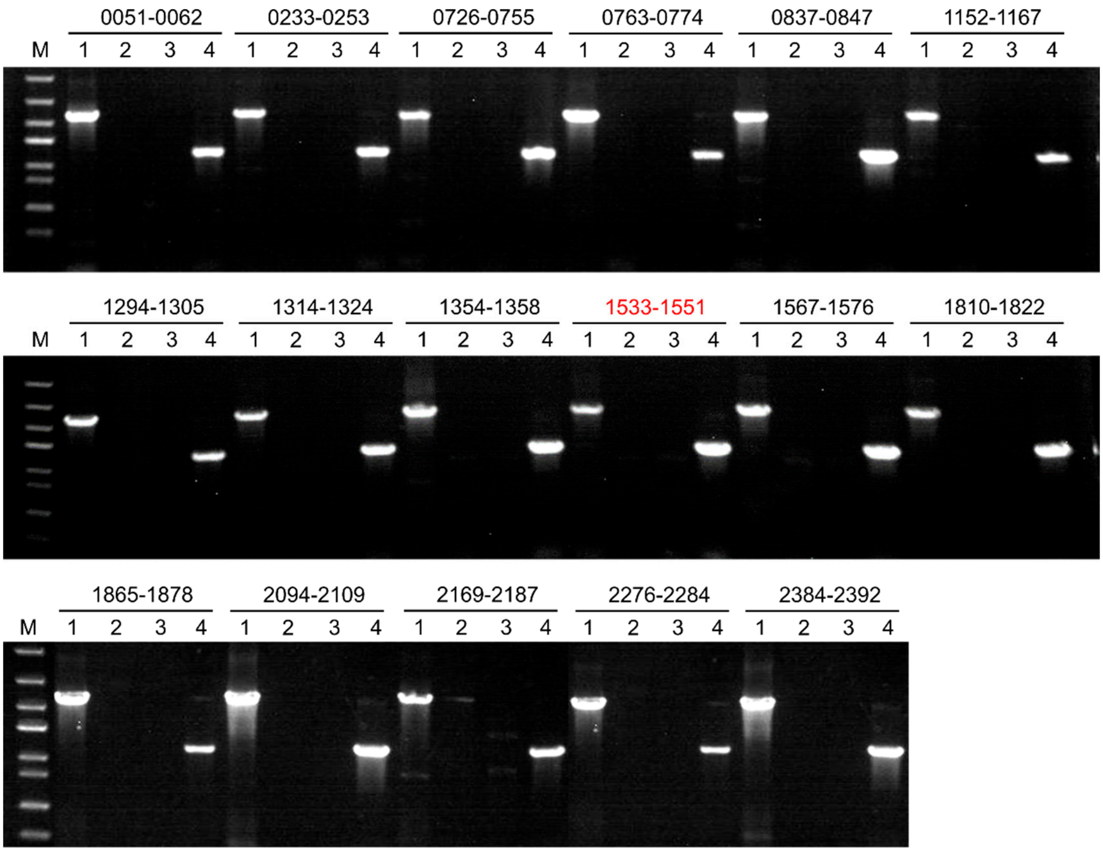

Figure S2. PCR examinations of the single-deletion mutants generated by using the editing plasmids derived from Cpf1b-Sp. Region 1533-1551, in addition to the 19 identified nonessential regions in Figure S1, was also completely deleted (numbers in red). DNA markers (M) are the same as those in Figure S1. The short dash-linked numbers on the top stand for the deleted regions, indicated by the first and the last ORFs. The templates for lanes 1 and 3 are the genomic DNA of single mutants, and for lanes 2 and 4 are the genomic DNA of WT. The primers for lanes 1 and 2 are deleted region-F′/R′ (For example, primers 0051-0062-F′/R′ for region 0051-0062), and for lanes 3 and 4 are deleted region-F′/2 (For example, primers 0051-0062-F′/2 for region 0051-0062).

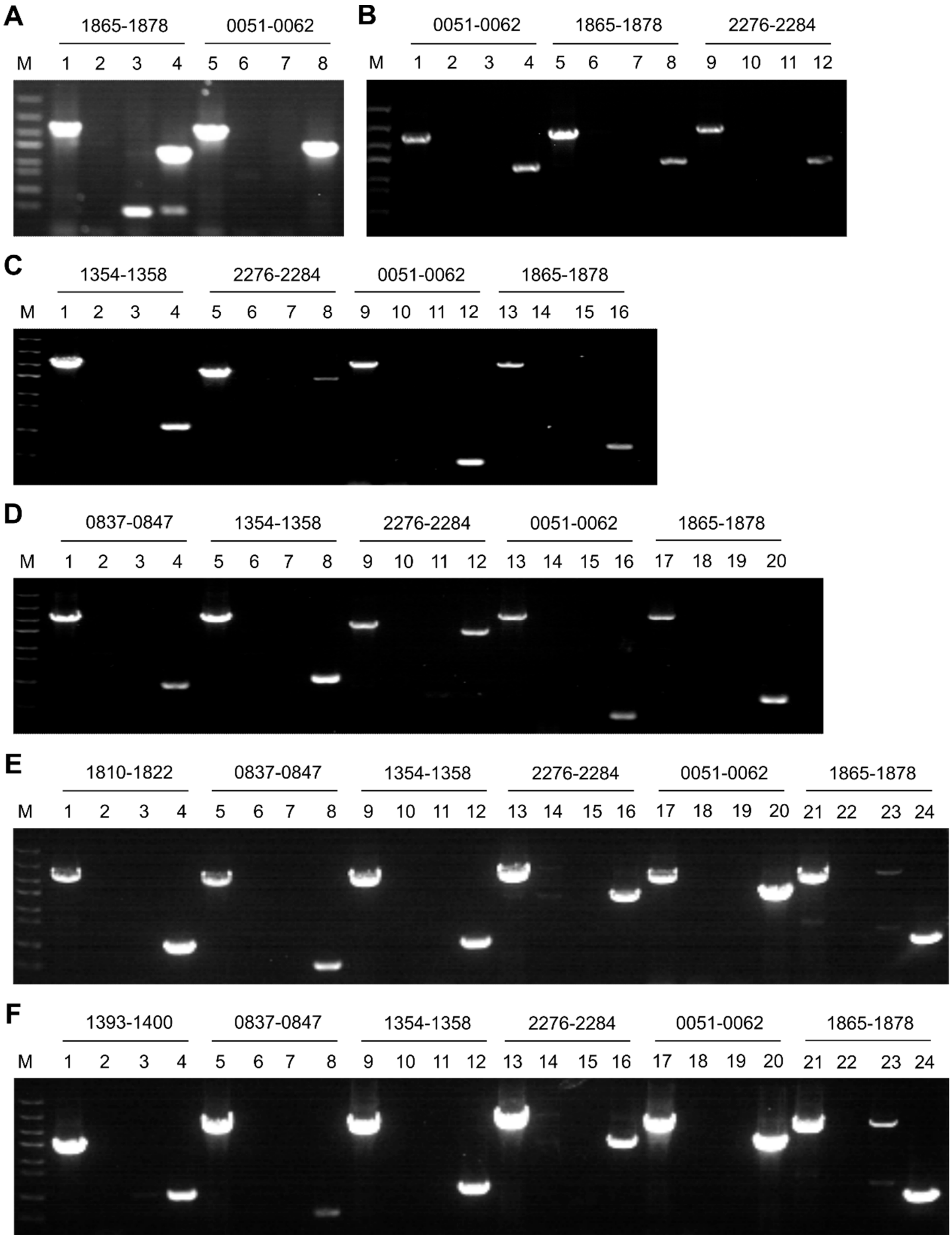

Figure S3. PCR examinations of multiple-deletion mutants. DNA markers (M) are the same as those in Figure S1. The short dash-linked numbers on the top stand for the deleted regions, indicated by the first and the last ORFs (For example, 0051-0062 for Synpcc7942_0051 ~ Synpcc7942_0062). (**A**) Genotype analysis of the double mutant (*Δ1865-1878/0051-0062*). The templates for lanes 1, 3, 5 and 7 are the genomes of the double mutant, and for lanes 2, 4, 6 and 8 are the genomes of the WT. The primers for PCR are Cr-1865-F′/R′ (lanes 1 and 2), Cr-1865-F/R (lanes 3 and 4), Cr-0051-F′/R′ (lanes 5 and 6), Cr-0051-F/R (lanes 7 and 8). (**B**) Genotype analysis of the triple mutant *b* (*Δ0051-0062/1865-1878/2276-2284*). The templates for lanes 1, 3, 5, 7, 9 and 11 are

the genomes of the triple mutant *B*, and for lanes 2, 4, 6, 8, 10 and 12 are the genomes of the WT. The primers for PCR are 0051-0062-F′/R′ (lanes 1 and 2), 0051-0062-F′/2 (lanes 3 and 4), 1865-1878-F′/R′ (lanes 5 and 6), 1865-1878-F′/2 (lanes 7 and 8), 2276-2284-F′/R′ (lanes 9 and 10), 2276-2284-F′/2 (lanes 11 and 12). (**C**) Genotype analysis of the quadruple mutant *c* (*Δ1354-1358/2276-2284/0051-0062/1865-1878*). The templates for lanes 1, 3, 5, 7, 9, 11, 13 and 15 are the genomes of the quadruple mutant *c*, and for lanes 2, 4, 6, 8, 10, 12, 14 and 16 are the genomes of the WT. The primers for PCR are Cr-1354-F′/R′ (lanes 1 and 2), Cr-1354-F/R-1 (lanes 3 and 4), Cr-2276-F′-1/R′ (lanes 5 and 6), Cr-2276-F/R (lanes 7 and 8), Cr-0051-F′/R′ (lanes 9 and 10), Cr-0051-F/R-1 (lanes 11 and 12), Cr-1865-F′/R′ (lanes 13 and 14), Cr-1865-F-1/R-1 (lanes 15 and 16). (**D**) Genotype analysis of the quintuple mutant *d* (*Δ0837-0847/1354-1358/2276-2284/0051-0062/1865-1878*). The templates for lanes 1, 3, 5, 7, 9, 11, 13, 15, 17 and 19 are the genomes of mutant *d*, and for lanes 2, 4, 6, 8, 10, 12, 14, 16, 18 and 20 are the genomes of the WT. The primers for PCR are Cr-0837-F′/R′ (lanes 1 and 2), Cr-0837-F-1/R-1 (lanes 3 and 4), Cr-1354-F′/R′ (lanes 5 and 6), Cr-1354-F/R-1 (lanes 7 and 8), Cr-2276-F′-1/R′ (lanes 9 and 10), Cr-2276-F/R (lanes 11 and 12), Cr-0051-F′/R′ (lanes 13 and 14), Cr-0051-F/R-1 (lanes 15 and 16), Cr-1865-F′/R′ (lanes 17 and 18), Cr-1865-F-1/R-1 (lanes 19 and 20). (**E**) Genotype analysis of the sextuple mutant *e1* (*Δ1810-1822/0837-0847/1354-1358/2276-2284/0051-0062/1865-1878*). The templates for lanes 1, 3, 5, 7, 9, 11, 13, 15, 17, 19, 21 and 23 are the genomes of the sextuple mutant *e1*, and for lanes 2, 4, 6, 8, 10, 12, 14, 16, 18, 20, 22 and 24 are the genomes of the WT. The primers for PCR are Cr-1810-F′/R′ (lanes 1 and 2), Cr-1810-F-1/R-1 (lanes 3 and 4), Cr-0837-F′/R′ (lanes 5 and 6), Cr-0837-F-3/R-3(lanes 7 and 8), Cr-1354-F′/R′ (lanes 9 and 10), Cr-1354-F/R-1 (lanes 11 and 12), Cr-2276-F′/R′ (lanes 13 and 14), Cr-2276-F/R (lanes 15 and 16), Cr-0051-F′/R′ (lanes 17 and 18), Cr-0051-F/R (lanes 19 and 20), Cr-1865-F′/R′ (lanes 21 and 22), Cr-1865-F/R-2 (lanes 23 and 24). (**F**) Genotype analysis of the sextuple mutant *e2* (*Δ1393-1400/0837-0847/1354-1358/2276-2284/0051-0062/1865-1878*). The templates for lanes 1, 3, 5, 7, 9, 11, 13, 15, 17, 19, 21 and 23 are the genomes of the sextuple mutant *e2*, and for lanes 2, 4, 6, 8, 10, 12, 14, 16, 18, 20, 22 and 24 are the genomes of the WT. The primers for PCR are Cr-1393-F′-1/R′-1 (lanes 1 and 2), Cr-1393-F-1/R-1 (lanes 3 and 4), Cr-0837-F′/R′ (lanes 5 and 6), Cr-0837-F-3/R-3(lanes 7 and 8), Cr-1354-F′/R′ (lanes 9 and 10), Cr-1354-F/R-1 (lanes 11 and 12), Cr-2276-F′/R′ (lanes 13 and 14), Cr-2276-F/R (lanes 15 and 16), Cr-0051-F′/R′ (lanes 17 and 18), Cr-0051-F/R (lanes 19 and 20), Cr-1865-F′/R′ (lanes 21 and 22), Cr-1865-F/R-2 (lanes 23 and 24).

Table S1. A list of wild-type and mutant strains generated in this study

Strains	Abbreviations	Fully segregated?	Descriptions
Synechococcus sp. PCC 7942	WT (*a*)		The wild type strain
Δ0051-0062		Yes	The genomic region 0051-0062* deleted in the WT, by using the editing plasmid pHB6588 or pHB6589
Δ0233-0253		Yes	The genomic region 0233-0253 deleted in the WT, by using the editing plasmid pHB6848 or pHB6818
Δ0363-0373		Yes	The genomic region 0363-0373 deleted in the WT, by using the editing plasmid pHB6856 or pHB6857
Δ0726-0755		Yes	The genomic region 0726-0755 deleted in the WT, by using the editing plasmid pHB6729 or pHB6730
Δ0763-0774		Yes	The genomic region 0763-0774 deleted in the WT, by using the editing plasmid pHB6881 or pHB6849
Δ0837-0847		Yes	The genomic region 0837-0847 deleted in the WT, by using the editing plasmid pHB6878 or pHB6879
Δ0854-0860		Yes	The genomic region 0854-0860 deleted in the WT, by using the editing plasmid pHB6880 or pHB6817
Δ1152-1167		Yes	The genomic region 1152-1167 deleted in the WT, by using the editing plasmid pHB6731 or pHB6732
Δ1294-1305		Yes	The genomic region 1294-1305 deleted in the WT, by using the editing plasmidpHB6884 or pHB6885
Δ1314-1324		Yes	The genomic region 1314-1324 deleted in the WT, by using the editing plasmidpHB6886 or pHB6887
Δ1354-1358		Yes	The genomic region 1354-1358 deleted in the WT, by using the editing plasmid pHB6858 or pHB6859
Δ1393-1400		Yes	The genomic region 1393-1400 deleted in the WT, by using the editing plasmid pHB6890 or pHB6891
Δ 1533-1551		No	The genomic region 1533-1551 deleted in the WT, by using the editing plasmid pHB6892 or pHB6893
Δ1567-1576		Yes	The genomic region 1567-1576 deleted in the WT, by using the editing plasmid pHB6894 or pHB6895
Δ1640-1650		No	The genomic region 1640-1650 deleted in the WT, by using the editing plasmid pHB6896 or pHB6897
Δ1810-1822		Yes	The genomic region 1810-1822 deleted in the WT, by using the editing plasmid pHB6898 or pHB6899
Δ1865-1878		Yes	The genomic region 1865-1878 deleted in the WT, by using the editing plasmid pHB6900 or pHB6901
Δ2094-2109		Yes	The genomic region 2094-2109 deleted in the WT, by using the editing plasmid pHB6846 or pHB6882
Δ2169-2187		Yes	The genomic region 2169-2187 deleted in the WT, by using the editing plasmid pHB6902 or pHB6903

Δ2276-2284		Yes	The genomic region 2276-2284 deleted in the WT, by using the editing plasmid pHB6883 or pHB6822
Δ2384-2392		Yes	The genomic region 2384-2392 deleted in the WT, by using the editing plasmid pHB6904 or pHB6905
Δ2476-2490		No	The genomic region 2476-2490 deleted in the WT, by using the editing plasmid pHB6850 or pHB6819
Δ2573-2578		No	The genomic region 2573-2578 deleted in the WT, by using the editing plasmid pHB6888 or pHB6889
Δ0051-0062/1865-1878		Yes	The genomic region 1865-1878 deleted in the *Δ0051-0062* single mutant, by using the editing plasmid pHB6900 or pHB6901
Δ0051-0062/1865-1878/2276-2284	triple (*b*)	Yes	The genomic region 2276-2284 deleted in the *Δ0051-0062/1865-1878*, by using the editing plasmid pHB6912
Δ1354-1358/2276-2284/0051-0062/1865-1878	quadruple (*c*)	Yes	The genomic region 1354-1358 deleted in the *Δ2276-2284/0051-0062/1865-1878*, by using the editing plasmid pHB6858
Δ0837-0847/1354-1358/2276-2284/0051-0062/1865-1878	quintuple (*d*)	Yes	The genomic region 0837-0847 deleted in the *Δ1354-1358/2276-2284/0051-0062/1865-1878*, by using the editing plasmid pHB6858
Δ1810-1822/0837-0847/1354-1358/2276-2284/0051-0062/1865-1878	sextuple (*e1*)	Yes	The genomic region 1810-1822 deleted in the *Δ0837-0847/1354-1358/2276-2284/0051-0062/1865-1878*, by using the editing plasmid pHB6858
Δ1393-1400/0837-0847/1354-1358/2276-2284/0051-0062/1865-1878	sextuple (*e2*)	Yes	The genomic region 1810-1822 deleted in the *Δ1393-1400/1354-1358/2276-2284/0051-0062/1865-1878*, by using the editing plasmid pHB6858
Δ0726-0755/1810-1822/0837-0847/1354-1358/2276-2284/0051-0062/1865-1878	septuple (*f*)	Yes	The genomic region 0726-0755 deleted in the *Δ1810-1827/0837-0847/1354-1358/2276-2284/0051-0062/1865-1878*, by using the editing plasmid pHB6858

*The genomic region to be deleted is denoted by the short dash-linked numbers of the first and last ORFs.

Table S2. Primers used in this study

Primers	Sequences（5′ -> 3′）	Descriptions
List of 69 pairs of primers for gRNA sequences		
gRNA-0051-F	AGATAATCGTAAGGGATGCTCATCTA	gRNA sequences in editing plasmids with Sp (spectinomycin) or Km (kanamycin) resistance
gRNA-0051-R	AGACTAGATGAGCATCCCTTACGATT	
gRNA-0854-F	AGATATCGATCGCGCTAAGGCAAAGA	
gRNA-0854-R	AGACTCTTTGCCTTAGCGCGATCGAT	
gRNA-1294-F	AGATTCGAAGCAGCACAGGCAAATGG	
gRNA-1294-R	AGACCCATTTGCCTGTGCTGCTTCGA	
gRNA-0232-F	AGATTGCCCTCGCTCAATAGTCAGTA	
gRNA-0232-R	AGACTACTGACTATTGAGCGAGGGCA	
gRNA-0726-F	AGATAATCCAGCTAGAATGCAGTCAC	
gRNA-0726-R	AGACGTGACTGCATTCTAGCTGGATT	
gRNA-0837-F	AGATACTTAGGCATGGAGATAGTCCT	
gRNA-0837-R	AGACAGGACTATCTCCATGCCTAAGT	
gRNA-1152-F	AGATGCAAGCAACCTATGACGAATTC	
gRNA-1152-R	AGACGAATTCGTCATAGGTTGCTTGC	
gRNA-0763-F	AGATCAGAACGGCAAGTTCATCAATG	
gRNA-0763-R	AGACCATTGATGAACTTGCCGTTCTG	
gRNA-1314-F	AGATCAAGTCAAAGCGATCGCAGACC	
gRNA-1314-R	AGACGGTCTGCGATCGCTTTGACTTG	
gRNA-1354-F	AGATAGTGGGTAAGCTTCCTAGCGCT	
gRNA-1354-R	AGACAGCGCTAGGAAGCTTACCCACT	
gRNA-1393-F	AGATGGGAAACGCATGAAGCAATTAA	
gRNA-1393-R	AGACTTAATTGCTTCATGCGTTTCCC	
gRNA-1533-F	AGATGTTCATGGCTTCCATCGCATCG	
gRNA-1533-R	AGACCGATGCGATGGAAGCCATGAAC	
gRNA-1567-F	AGATAATGTCCCACTGGCTCAGGACA	
gRNA-1567-R	AGACTGTCCTGAGCCAGTGGGACATT	
gRNA-1640-F	AGATCATAGACCGCAAATCGCTGTGG	
gRNA-1640-R	AGACCCACAGCGATTTGCGGTCTATG	
gRNA-1810-F	AGATCAGTTAGCAACTGACTTTCAGC	
gRNA-1810-R	AGACGCTGAAAGTCAGTTGCTAACTG	
gRNA-1865-F	AGATGTGACTTCTTCCACGGCGATTT	
gRNA-1865-R	AGACAAATCGCCGTGGAAGAAGTCAC	
gRNA-2094-F	AGATGCATCATGACCTATGAAGGGAT	
gRNA-2094-R	AGACATCCCTTCATAGGTCATGATGC	
gRNA-2169-F	AGATAGCCGCAATCCAAACAGCAATC	
gRNA-2169-R	AGACGATTGCTGTTTGGATTGCGGCT	
gRNA-2276-F	AGATCCCTACACGGCGAAGATGCCTA	
gRNA-2276-R	AGACTAGGCATCTTCGCCGTGTAGGG	
gRNA-2384-F	AGATCAGGCTGAAGATCCACTTCCCA	

gRNA-2384-R	AGACTGGGAAGTGGATCTTCAGCCTG	
gRNA-2476-F	AGATCGGCTGATGACAGTTCGCGATC	
gRNA-2476-R	AGACGATCGCGAACTGTCATCAGCCG	
gRNA-2573-F	AGATAAGGCTGCATAGAGGAGAGTGC	
gRNA-2573-R	AGACGCACTCTCCTCTATGCAGCCTT	
gRNA-0363-F	AGATCATGTCTCAACATCTGGAAACA	
gRNA-0363-R	AGACTGTTTCCAGATGTTGAGACATG	
AarI-F	AGGCAAAAACGGGTCTAAGAAC	Verification of gRNA cloning into the vector
AarI-R	GGAATAGTAGCATCTACAACAGTAG	
gRNA-0051-F2	AGATGTCACTGACGAGATGCAATAGC	
gRNA-0051-R2	AGACGCTATTGCATCTCGTCAGTGAC	
gRNA-0232-F1	AGATAGGGTGCGAATGGCTTCTGCCA	
gRNA-0232-R1	AGACTGGCAGAAGCCATTCGCACCCT	
gRNA-0232-F2	AGATTGCCCTCGCTCAATAGTCAGTA	
gRNA-0232-R2	AGACTACTGACTATTGAGCGAGGGCA	
gRNA-0363-F1	AGATTGTCAACAGTTCGCGATCGCCT	
gRNA-0363-R1	AGACAGGCGATCGCGAACTGTTGACA	
gRNA-0726-F1	AGATACCTTCTGGCTCGCTACGATCG	
gRNA-0726-R1	AGACCGATCGTAGCGAGCCAGAAGGT	
gRNA-0763-F2	AGATATCGCGCGATCATCTCATGCCA	
gRNA-0763-R2	AGACTGGCATGAGATGATCGCGCGAT	
gRNA-0837-F2	AGATAGATGAGAACCGAGCTAAAGGC	
gRNA-0837-R2	AGACGCCTTTAGCTCGGTTCTCATCT	
gRNA-0854-F2	AGATAAACGAGTTCTCTCTCTGGCCC	
gRNA-0854-R2	AGACGGGCCAGAGAGAGAACTCGTTT	
gRNA-1152-F2	AGATGAAAGATGCATCAGGGAAGAGA	gRNA sequences in editing plasmids with Km resistance
gRNA-1152-R2	AGACTCTCTTCCCTGATGCATCTTTC	
gRNA-1294-F1	AGATCAGGACATGTTAGGCTGTAAGA	
gRNA-1294-R1	AGACTCTTACAGCCTAACATGTCCTG	
gRNA-1314-F1	AGATCACCGTGGTTCTGGTCGATGAC	
gRNA-1314-R1	AGACGTCATCGACCAGAACCACGGTG	
gRNA-1354-F1	AGATGCAACTCAACGATATCTAGCCA	
gRNA-1354-R1	AGACTGGCTAGATATCGTTGAGTTGC	
gRNA-1393-F1	AGATGGTCTGGAGCCTCAGCTGTGAT	
gRNA-1393-R1	AGACATCACAGCTGAGGCTCCAGACC	
gRNA-1533-F2	AGATTGCTAACTCTGTCAATCGCCCA	
gRNA-1533-R2	AGACTGGGCGATTGACAGAGTTAGCA	
gRNA-1567-F1	AGATTTTCTGGACAGGTAGACTTTCA	
gRNA-1567-R1	AGACTGAAAGTCTACCTGTCCAGAAA	
gRNA-1640-F2	AGATCCAAGGATTTAGGGCGAAACCG	
gRNA-1640-R2	AGACCGGTTTCGCCCTAAATCCTTGG	
gRNA-1810-F2	AGATCAGTCTGGACACCTGAAATTCT	
gRNA-1810-R2	AGACAGAATTTCAGGTGTCCAGACTG	

gRNA-1865-F1	AGATAGGCTACGATCGACTGGTGTTC	
gRNA-1865-R1	AGACGAACACCAGTCGATCGTAGCCT	
gRNA-2094-F2	AGATGCTCAAACTCAACTGACGCCCC	
gRNA-2094-R2	AGACGGGGCGTCAGTTGAGTTTGAGC	
gRNA-2169-F1	AGATCAAGGTGGTGGTGCGATCGTAG	
gRNA-2169-R1	AGACCTACGATCGCACCACCACCTTG	
gRNA-2276-F2	AGATCACCCCAACAAGCCCTCCTG	
gRNA-2276-R2	AGACCAGGAGGGCTTGTTGGGGTG	
gRNA-2384-F2	AGATTCAGTGTGACTCGTGTTCGTCC	
gRNA-2384-R2	AGACGGACGAACACGAGTCACACTGA	
gRNA-2476-F1	GATTGGCAGGGATTCTAGCCGCGAT	
gRNA-2476-R1	AGACATCGCGGCTAGAATCCCTGCCA	
gRNA-2573-F1	AGATTAGTACGTGTGTAGCCCAGGAT	
gRNA-2573-R1	AGACATCCTGGGCTACACACGTACTA	
Primers used in construction of editing plasmids		
0051-0062-F1	GCAGAAATTCGATATCTAGATCTGGCGCTCAACTTCTCCTGTAAGGC	Amplification of the sequence upstream of the deletion region 0051-0062
0051-0062-R1	CGTCAGGCTGAGTAGAGGTTCGGGAGAATCACTCACGGGATGCG	
0051-0062-F2	CGCATCCCGTGAGTGATTCTCCCGAACCTCTACTCAGCCTGACG	Amplification of the sequence downstream of the deletion region 0051-0062
0051-0062-R2	CGCAACGTTGTTGCCATTGCGGATCCCTCGCTATCGCTTCGCTGCCC	
0232-0253-F1	TGGCAGAAATTCGATATCTAGCCAGTCTTCCTGGGACATCTAGAC	Amplification of the sequence upstream of the deletion region 0232-0253
0232-0253-R1	CGGTAGTAAGTCAGCCAATCGTGACGCGGCATTCCCCTTGGAGATGGTAG	
0232-0253-F2	CTACCATCTCCAAGGGGAATGCCGCGTCACGATTGGCTGACTTACTACCG	Amplification of the sequence downstream of the deletion region 0232-0253
0232-0253-R2	GCAACGTTGTTGCCATTGCGCAATGACTTTCAGCCCTTGAGGACTTTG	
0363-0373-F1	TGGCAGAAATTCGATATCTAGCCCATGACACTACCGATTCATC	Amplification of the sequence upstream of the deletion region 0363-0373
0363-0373-R1	CCTACCGATCCAACTCCCTCTACGCAACCAACAAGAGCCTGGCCTC	
0363-0373-F2	GAGGCCAGGCTCTTGTTGGTTGCGTAGAGGGAGTTGGATCGGTAGG	Amplification of the sequence downstream of the deletion region 0363-0373
0363-0373-R2	GCAACGTTGTTGCCATTGCGGTGATGCAGAACCATTGTGGTG	
0726-0755-F1	TGGCAGAAATTCGATATCTAGAGAACGCTGATCCTTGTGCACC	Amplification of the sequence upstream of the deletion region 0726-0755
0726-0755-R1	CTTGTGCTGGAGGATTTGAGACATGGCGCGCTTCGACGACGTAGCGATG	

0726-0755-F2	CATCGCTACGTCGTCGAAGCGCGCCATGT CTCAAATCCTCCAGCACAAG	Amplification of the sequence downstream of the deletion region 0726-0755
0726-0755-R2	GCAACGTTGTTGCCATTGCGCGTCTTTCCA TCTACGTCATGAATCAC	
0763-0774-F1	TGGCAGAAATTCGATATCTAGTGAAATTC CCTCAGACGTCCTACTC	Amplification of the sequence upstream of the deletion region 0763-0774
0763-0774-R1	GGTTTGCTCCAGCATGGCTAAATGCGCTA CCTAAGAGAACTGATGGAAGAGCG	
0763-0774-F2	CGCTCTTCCATCAGTTCTCTTAGGTAGCGC ATTTAGCCATGCTGGAGCAAACC	Amplification of the sequence downstream of the deletion region 0763-0774
0763-0774-R2	GCAACGTTGTTGCCATTGCGCAAGTGATC GACGACATCTTGGACATC	
0837-0847-F1	TGGCAGAAATTCGATATCTAGCCTATTGT CCCACTTGATCGAGAACT	Amplification of the sequence upstream of the deletion region 0837-0847
0837-0847-R1	CTGCGAGAACTGAGCGACAGCATTGGGA ACTGATGGCGCAGTTAGCCG	
0837-0847-F2	CGGCTAACTGCGCCATCAGTTCCCAATGC TGTCGCTCAGTTCTCGCAC	Amplification of the sequence downstream of the deletion region 0837-0847
0837-0847-R2	GCAACGTTGTTGCCATTGCGGGACAGTCT TGAACCCAAGCCTAC	
0854-0860-F1	TGGCAGAAATTCGATATCTAGTCTGTAAT GTCGTCGGCACGCCC	Amplification of the sequence upstream of the deletion region 0854-0860
0854-0860-R1	GATAATGAGCTGATCGGACCTCTCCTCCA GTCCTTCTGCCTCAGCAATG	
0854-0860-F2	CATTGCTGAGGCAGAAGGACTGGAGGAG AGGTCCGATCAGCTCATTATC	Amplification of the sequence downstream of the deletion region 0854-0860
0854-0860-R2	GCAACGTTGTTGCCATTGCGTGAATCGCA CCTACCATTGGCGTC	
1152-1167-F1	TGGCAGAAATTCGATATCTAGAATCACCC GCAGTTGCTGAGCATCC	Amplification of the sequence upstream of the deletion region 1152-1167
1152-1167-R1	GAAGTCAGCCAAGAACTGAACATGCCAG GTCGTCAGCACCACAGTTGG	
1152-1167-F2	CCAACTGTGGTGCTGACGACCTGGCATGT TCAGTTCTTGGCTGACTTC	Amplification of the sequence downstream of the deletion region 1152-1167
1152-1167-R2	GCAACGTTGTTGCCATTGCGGGCACTACA AACCCTCCTAGAAGG	
1294-1305-F1	TGGCAGAAATTCGATATCTACCAAGGCGT GATTCATCGGGAC	Amplification of the sequence upstream of the deletion region 1294-1305
1294-1305-R1	GATCCGATGACGCGCCTCGATCCGGTTAG CCCCGCAAAGATTCGTGCC	
1294-1305-F2	GGCACGAATCTTTGCGGGGCTAACCGGAT CGAGGCGCGTCATCGGATC	Amplification of the sequence downstream of the deletion region 1294-1305
1294-1305-R2	GCAACGTTGTTGCCATTGCGGTTGCCAAC GAGGCTTTGCATGCG	

1314-1324-F1	TGGCAGAAATTCGATATCTAGGATGTTTC CGATGTCGTTGCGGAC	Amplification of the sequence upstream of the deletion region 1314-1324
1314-1324-R1	GCGGGAAATTCCTGAGATTCCCTCGAAGC CCTCCTTTGCAGATAGCG	
1314-1324-F2	CGCTATCTGCAAAGGAGGGCTTCGAGGGA ATCTCAGGAATTTCCCGC	Amplification of the sequence downstream of the deletion region 1314-1324
1314-1324-R2	GCAACGTTGTTGCCATTGCGCGGTCTCAG CAGACGAGTTTATCG	
1354-1358-F1	TGGCAGAAATTCGATATCTAGTAGTTGAT TGATCTGCGATGACAG	Amplification of the sequence upstream of the deletion region 1354-1358
1354-1358-R1	CTCTTAAGCAAACCCTTGGTTCGGCGTTA TCGATGACGACTGGTCCCGC	
1354-1358-F2	GCGGGACCAGTCGTCATCGATAACGCCGA ACCAAGGGTTTGCTTAAGAG	Amplification of the sequence downstream of the deletion region 1354-1358
1354-1358-R2	GCAACGTTGTTGCCATTGCGCGGAGTTCA TCAGTCGATGACGGC	
1393-1400-F1	TGGCAGAAATTCGATATCTACCCTGTACT TGCGGCCTGAATC	Amplification of the sequence upstream of the deletion region 1393-1400
1393-1400-R1	GGTCATACGCTGAGGAGACGCCAGGAGA GTGCTGGCCAAGCCTTCTGGG	
1393-1400-F2	CCCAGAAGGCTTGGCCAGCACTCTCCTGG CGTCTCCTCAGCGTATGACC	Amplification of the sequence downstream of the deletion region 1393-1400
1393-1400-R2	GCAACGTTGTTGCCATTGCGGGGGCTCAT TGTTAATCGTTCTCGAC	
1533-1551-F1	TGGCAGAAATTCGATATCTACTAAACTGG CGCACTTATCGCTGC	Amplification of the sequence upstream of the deletion region 1533-1551
1533-1551-R1	CTAGCCCTGAGTGGTTAGCAGCTCCGGCC TCTCTTGAGTGAACCTGCC	
1533-1551-F2	GGCAGGTTCACTCAAGAGAGGCCGGAGC TGCTAACCACTCAGGGCTAG	Amplification of the sequence downstream of the deletion region 1533-1551
1533-1551-R2	GCAACGTTGTTGCCATTGCGGTGGCCATC ATTGGCTATGGCTCGC	
1567-1576-F1	TGGCAGAAATTCGATATCTACAACAGATC CCCACGTGCTGGCTATC	Amplification of the sequence upstream of the deletion region 1567-1576
1567-1576-R1	GGGTCAAAATGCCAGCGAGAAATTTCTGG GATCTACCGGACAGGTGTGGC	
1567-1576-F2	GCCACACCTGTCCGGTAGATCCCAGAAAT TTCTCGCTGGCATTTTGACCC	Amplification of the sequence downstream of the deletion region 1567-1576
1567-1576-R2	GCAACGTTGTTGCCATTGCGGCTAAAGAT CGATCGCCCCATTCTGAG	
1640-1650-F1	TGGCAGAAATTCGATATCTAGCCAAGCGT ATTGGAGCTGCAC	Amplification of the sequence upstream of the deletion region 1640-1650
1640-1650-R1	GATCAGTCTTCCGTAATTCACGTCATGGT GTGACACTTCCGTCACAATCCTC	

1640-1650-F2	GAGGATTGTGACGGAAGTGTCACACCATGACGTGAATTACGGAAGACTGATC	Amplification of the sequence downstream of the deletion region 1640-1650
1640-1650-R2	GCAACGTTGTTGCCATTGCGGTAGGCGCAGATACCAAACCACCC	
1810-1822-F1	TGGCAGAAATTCGATATCTACAGATCGAAGGCGATTTTCAGTACTAC	Amplification of the sequence upstream of the deletion region 1810-1822
1810-1822-R1	GCAAGACAAAGCGGCAGCTGTAGCGTGATCGCTGCAATCCATGCGGCTG	
1810-1822-F2	CAGCCGCATGGATTGCAGCGATCACGCTACAGCTGCCGCTTTGTCTTGC	Amplification of the sequence downstream of the deletion region 1810-1822
1810-1822-R2	GCAACGTTGTTGCCATTGCGGATTCGCATCTTGGCAGACAAGCAC	
1865-1878-F1	TGGCAGAAATTCGATATCTAGCGGCGCCTGACTATTTGCGATCG	Amplification of the sequence upstream of the deletion region 1865-1878
1865-1878-R1	CCGCTATGCGGAATCCTTGCTGGCTTGAACGCAGGTATAGCCTCAGC	
1865-1878-F2	GCTGAGGCTATACCTGCGTTCAAGCCAGCAAGGATTCCGCATAGCGG	Amplification of the sequence downstream of the deletion region 1865-1878
1865-1878-R2	GCAACGTTGTTGCCATTGCGGAGGGAGAAGGATCAGCGACCATAG	
2094-2109-F1	TGGCAGAAATTCGATATCTACATCTCGCAGGAGTATCTCGATCGC	Amplification of the sequence upstream of the deletion region 2094-2109
2094-2109-R1	CGTTCGCCGGTTTGCACTTTCCAGGCAAGCCTGGGTGATAAGAAGTCC	
2094-2109-F2	GGACTTCTTATCACCCAGGCTTGCCTGGAAAGTGCAAACCGGCGAACG	Amplification of the sequence downstream of the deletion region 2094-2109
2094-2109-R2	GCAACGTTGTTGCCATTGCGCCCAATTCGCGGTTCCAACTTTGC	
2169-2187-F1	TGGCAGAAATTCGATATCTAGATATCTTGGCCTATCTGCGGGCG	Amplification of the sequence upstream of the deletion region 2169-2187
2169-2187-R1	CGTCCCATCTGAGGAGATGCACCGCCCGTTGTCTTAGACAGCAGG	
2169-2187-F2	CCTGCTGTCTAAGACAACGGGCGGTGCATCTCCTCAGATGGGACG	Amplification of the sequence downstream of the deletion region 2169-2187
2169-2187-R2	GCAACGTTGTTGCCATTGCGGGTGCGTTGCCAAGCTGATCGC	
2276-2284-F1	TGGCAGAAATTCGATATCTAGCGGTCAGTACTACCACTTGC	Amplification of the sequence upstream of the deletion region 2276-2284
2276-2284-R1	GCGTTTAGAAGAACTGCAAGGCACCGTGACGGCTCAAGGACTAGCGATTC	
2276-2284-F2	GAATCGCTAGTCCTTGAGCCGTCACGGTGCCTTGCAGTTCTTCTAAACGC	Amplification of the sequence downstream of the deletion region 2276-2284
2276-2284-R2	GCAACGTTGTTGCCATTGCGCGATCGCTGTATTGCAGCATTTAGCG	

2384-2392-F1	TGGCAGAAATTCGATATCTACAACTCGCAGATGCCAGTAATGACC	Amplification of the sequence upstream of the deletion region 2384-2392
2384-2392-R1	GACTCTTTACCGTTGTGGGATACTCCGAACAGAGCAACGCACAGCCTGTC	
2384-2392-F2	GACAGGCTGTGCGTTGCTCTGTTCGGAGTATCCCACAACGGTAAAGAGTC	Amplification of the sequence downstream of the deletion region 2384-2392
2384-2392-R2	GCAACGTTGTTGCCATTGCGCCAACAACGGGCTTCAGTTCTCGATC	
2476-2490-F1	TGGCAGAAATTCGATATCTAGGCACATCATCTCCATGCCTACTTAG	Amplification of the sequence upstream of the deletion region 2476-2490
2476-2490-R1	CTATGTCAGTGGCGATCGCCTGACGCAAACTCCTACCGAAACAGAGACC	
2476-2490-F2	GGTCTCTGTTTCGGTAGGAGTTTGCGTCAGGCGATCGCCACTGACATAG	Amplification of the sequence downstream of the deletion region 2476-2490
2476-2490-R2	GCAACGTTGTTGCCATTGCGGACACCTTATAACCGCCACCGCC	
2573-2578-F1	TGGCAGAAATTCGATATCTACGGAGTCGAAGAATCCCATAGCG	Amplification of the sequence upstream of the deletion region 2573-2578
2573-2578-R1	GCAGGACTTTCAGGCTCCCATTGGGATATAACTCGGTACGAGGCAGC	
2573-2578-F2	GCTGCCTCGTACCGAGTTATATCCCAATGGGAGCCTGAAAGTCCTGC	Amplification of the sequence downstream of the deletion region 2573-2578
2573-2578-R2	GCAACGTTGTTGCCATTGCGGGATCAGGCTGCAATTAAAGCAGCC	
Cr-0051-F1	GCAGAAATTCGATATCTAGATCTGGCGCTCAACTTCTCCTGTAAGGC	Amplification of the sequence upstream of the deletion region 0051-0062
Cr-0051-R1	CGTCAGGCTGAGTAGAGGTTCGGGAGAATCACTCACGGGATGCG	
Cr-0051-F2	CGCATCCCGTGAGTGATTCTCCCGAACCTCTACTCAGCCTGACG	Amplification of the sequence downstream of the deletion region 0051-0062
Cr-0051-R2	CGCAACGTTGTTGCCATTGCGGATCCCTCGCTATCGCTTCGCTGCCC	
Cr-0253-F1	TGGCAGAAATTCGATATCTAGCCAGTCTTCCTGGGACATCTAGAC	Amplification of the sequence upstream of the deletion region 0232-0253
Cr-0253-R1	CGGTAGTAAGTCAGCCAATCGTGACGCGGCATTCCCCTTGGAGATGGTAG	
Cr-0253-F2	CTACCATCTCCAAGGGGAATGCCGCGTCACGATTGGCTGACTTACTACCG	Amplification of the sequence downstream of the deletion region 0232-0253
Cr-0253-R2	GCAACGTTGTTGCCATTGCGCAATGACTTTCAGCCCTTGAGGACTTTG	
Cr-0363-F1	TGGCAGAAATTCGATATCTAGCCCATGACACTACCGATTCATC	Amplification of the sequence upstream of the deletion region 0363-0373
Cr-0363-R1	CCTACCGATCCAACTCCCTCTACGCAACCAACAAGAGCCTGGCCTC	

Cr-0363-F2	GAGGCCAGGCTCTTGTTGGTTGCGTAGAGGGAGTTGGATCGGTAGG	Amplification of the sequence downstream of the deletion region 0363-0373
Cr-0363-R2	GCAACGTTGTTGCCATTGCGGTGATGCAGAACCATTGTGGTG	
Cr-0726-F1	TGGCAGAAATTCGATATCTAGAGAACGCTGATCCTTGTGCACC	Amplification of the sequence upstream of the deletion region 0726-0755
Cr-0726-R1	CTTGTGCTGGAGGATTTGAGACATGGCGCGCTTCGACGACGTAGCGATG	
Cr-0726-F2	CATCGCTACGTCGTCGAAGCGCGCCATGTCTCAAATCCTCCAGCACAAG	Amplification of the sequence downstream of the deletion region 0726-0755
Cr-0726-R2	GCAACGTTGTTGCCATTGCGCGTCTTTCCATCTACGTCATGAATCAC	
Cr-0763-F1	TGGCAGAAATTCGATATCTAGTGAAATTCCCTCAGACGTCCTACTC	Amplification of the sequence upstream of the deletion region 0763-0774
Cr-0763-R1	GGTTTGCTCCAGCATGGCTAAATGCGCTACCTAAGAGAACTGATGGAAGAGCG	
Cr-0763-F2	CGCTCTTCCATCAGTTCTCTTAGGTAGCGCATTTAGCCATGCTGGAGCAAACC	Amplification of the sequence downstream of the deletion region 0763-0774
Cr-0763-R2	GCAACGTTGTTGCCATTGCGCAAGTGATCGACGACATCTTGGACATC	
Cr-0837-F1	TGGCAGAAATTCGATATCTAGCCTATTGTCCCACTTGATCGAGAACT	Amplification of the sequence upstream of the deletion region 0837-0847
Cr-0837-R1	CTGCGAGAACTGAGCGACAGCATTGGGAACTGATGGCGCAGTTAGCCG	
Cr-0837-F2	CGGCTAACTGCGCCATCAGTTCCCAATGCTGTCGCTCAGTTCTCGCAC	Amplification of the sequence downstream of the deletion region 0837-0847
Cr-0837-R2	GCAACGTTGTTGCCATTGCGGGACAGTCTTGAACCCAAGCCTAC	
Cr-0854-F1	TGGCAGAAATTCGATATCTAGTCTGTAATGTCGTCGGCACGCCC	Amplification of the sequence upstream of the deletion region 0854-0860
Cr-0854-R1	GATAATGAGCTGATCGGACCTCTCCTCCAGTCCTTCTGCCTCAGCAATG	
Cr-0854-F2	CATTGCTGAGGCAGAAGGACTGGAGGAGAGGTCCGATCAGCTCATTATC	Amplification of the sequence downstream of the deletion region 0854-0860
Cr-0854-R2	GCAACGTTGTTGCCATTGCGTGAATCGCACCTACCATTGGCGTC	
Cr-1152-F1	TGGCAGAAATTCGATATCTAGAATCACCCGCAGTTGCTGAGCATCC	Amplification of the sequence upstream of the deletion region 1152-1167
Cr-1152-R1	GAAGTCAGCCAAGAACTGAACATGCCAGGTCGTCAGCACCACAGTTGG	
Cr-1152-F2	CCAACTGTGGTGCTGACGACCTGGCATGTTCAGTTCTTGGCTGACTTC	Amplification of the sequence downstream of the deletion region 1152-1167
Cr-1152-R2	GCAACGTTGTTGCCATTGCGGGCACTACAAACCCTCCTAGAAGG	

Cr-1294-F1	TGGCAGAAATTCGATATCTACCAAGGCGTGATTCATCGGGAC	Amplification of the sequence upstream of the deletion region 1294-1305
Cr-1294-R1	GATCCGATGACGCGCCTCGATCCGGTTAGCCCCGCAAAGATTCGTGCC	
Cr-1294-F2	GGCACGAATCTTTGCGGGGCTAACCGGATCGAGGCGCGTCATCGGATC	Amplification of the sequence downstream of the deletion region 1294-1305
Cr-1294-R2	GCAACGTTGTTGCCATTGCGGTTGCCAACGAGGCTTTGCATGCG	
Cr-1314-F1	TGGCAGAAATTCGATATCTAGGATGTTTCCGATGTCGTTGCGGAC	Amplification of the sequence upstream of the deletion region 1314-1324
Cr-1314-R1	GCGGGAAATTCCTGAGATTCCCTCGAAGCCCTCCTTTGCAGATAGCG	
Cr-1314-F2	CGCTATCTGCAAAGGAGGGCTTCGAGGGAATCTCAGGAATTTCCCGC	Amplification of the sequence downstream of the deletion region 1314-1324
Cr-1314-R2	GCAACGTTGTTGCCATTGCGCGGTCTCAGCAGACGAGTTTATCG	
Cr-1354-F1	TGGCAGAAATTCGATATCTAGTAGTTGATTGATCTGCGATGACAG	Amplification of the sequence upstream of the deletion region 1354-1358
Cr-1354-R1	CTCTTAAGCAAACCCTTGGTTCGGCGTTATCGATGACGACTGGTCCCGC	
Cr-1354-F2	GCGGGACCAGTCGTCATCGATAACGCCGAACCAAGGGTTTGCTTAAGAG	Amplification of the sequence downstream of the deletion region 1354-1358
Cr-1354-R2	GCAACGTTGTTGCCATTGCGCGGAGTTCATCAGTCGATGACGGC	
Cr-1393-F1	TGGCAGAAATTCGATATCTACCCTGTACTTGCGGCCTGAATC	Amplification of the sequence upstream of the deletion region 1393-1400
Cr-1393-R1	GGTCATACGCTGAGGAGACGCCAGGAGAGTGCTGGCCAAGCCTTCTGGG	
Cr-1393-F2	CCCAGAAGGCTTGGCCAGCACTCTCCTGGCGTCTCCTCAGCGTATGACC	Amplification of the sequence downstream of the deletion region 1393-1400
Cr-1393-R2	GCAACGTTGTTGCCATTGCGGGGGCTCATTGTTAATCGTTCTCGAC	
Cr-1533-F1	TGGCAGAAATTCGATATCTACTAAACTGGCGCACTTATCGCTGC	Amplification of the sequence upstream of the deletion region 1533-1551
Cr-1533-R1	CTAGCCCTGAGTGGTTAGCAGCTCCGGCCTCTCTTGAGTGAACCTGCC	
Cr-1533-F2	GGCAGGTTCACTCAAGAGAGGCCGGAGCTGCTAACCACTCAGGGCTAG	Amplification of the sequence downstream of the deletion region 1533-1551
Cr-1533-R2	GCAACGTTGTTGCCATTGCGGTGGCCATCATTGGCTATGGCTCGC	
Cr-1567-F1	TGGCAGAAATTCGATATCTACAACAGATCCCCACGTGCTGGCTATC	Amplification of the sequence upstream of the deletion region 1567-1576
Cr-1567-R1	GGGTCAAAATGCCAGCGAGAAATTTCTGGGATCTACCGGACAGGTGTGGC	

Cr-1567-F2	GCCACACCTGTCCGGTAGATCCCAGAAATTTCTCGCTGGCATTTTGACCC	Amplification of the sequence downstream of the deletion region 1567-1576
Cr-1567-R2	GCAACGTTGTTGCCATTGCGGCTAAAGATCGATCGCCCCATTCTGAG	
Cr-1640-F1	TGGCAGAAATTCGATATCTAGCCAAGCGTATTGGAGCTGCAC	Amplification of the sequence upstream of the deletion region 1640-1650
Cr-1640-R1	GATCAGTCTTCCGTAATTCACGTCATGGTGTGACACTTCCGTCACAATCCTC	
Cr-1640-F2	GAGGATTGTGACGGAAGTGTCACACCATGACGTGAATTACGGAAGACTGATC	Amplification of the sequence downstream of the deletion region 1640-1650
Cr-1640-R2	GCAACGTTGTTGCCATTGCGGTAGGCGCAGATACCAAACCACCC	
Cr-1810-F1	TGGCAGAAATTCGATATCTACAGATCGAAGGCGATTTTCAGTACTAC	Amplification of the sequence upstream of the deletion region 1810-1822
Cr-1810-R1	GCAAGACAAAGCGGCAGCTGTAGCGTGATCGCTGCAATCCATGCGGCTG	
Cr-1810-F2	CAGCCGCATGGATTGCAGCGATCACGCTACAGCTGCCGCTTTGTCTTGC	Amplification of the sequence downstream of the deletion region 1810-1822
Cr-1810-R2	GCAACGTTGTTGCCATTGCGGATTCGCATCTTGGCAGACAAGCAC	
Cr-1865-F1	TGGCAGAAATTCGATATCTAGCGGCGCCTGACTATTTGCGATCG	Amplification of the sequence upstream of the deletion region 1865-1878
Cr-1865-R1	CCGCTATGCGGAATCCTTGCTGGCTTGAACGCAGGTATAGCCTCAGC	
Cr-1865-F2	GCTGAGGCTATACCTGCGTTCAAGCCAGCAAGGATTCCGCATAGCGG	Amplification of the sequence downstream of the deletion region 1865-1878
Cr-1865-R2	GCAACGTTGTTGCCATTGCGGAGGGAGAAGGATCAGCGACCATAG	
Cr-2094-F1	TGGCAGAAATTCGATATCTACATCTCGCAGGAGTATCTCGATCGC	Amplification of the sequence upstream of the deletion region 2094-2109
Cr-2094-R1	CGTTCGCCGGTTTGCACTTTCCAGGCAAGCCTGGGTGATAAGAAGTCC	
Cr-2094-F2	GGACTTCTTATCACCCAGGCTTGCCTGGAAAGTGCAAACCGGCGAACG	Amplification of the sequence downstream of the deletion region 2094-2109
Cr-2094-R2	GCAACGTTGTTGCCATTGCGCCCAATTCGCGGTTCCAACTTTGC	
Cr-2169-F1	TGGCAGAAATTCGATATCTAGATATCTTGGCCTATCTGCGGGCG	Amplification of the sequence upstream of the deletion region 2169-2187
Cr-2169-R1	CGTCCCATCTGAGGAGATGCACCGCCCGTTGTCTTAGACAGCAGG	
Cr-2169-F2	CCTGCTGTCTAAGACAACGGGCGGTGCATCTCCTCAGATGGGACG	Amplification of the sequence downstream of the deletion region 2169-2187
Cr-2169-R2	GCAACGTTGTTGCCATTGCGGGTGCGTTGCCAAGCTGATCGC	

Cr-2276-F1	TGGCAGAAATTCGATATCTAGCGGTCAGTACTACCACTTGC	Amplification of the sequence upstream of the deletion region 2276-2284
Cr-2276-R1	GCGTTTAGAAGAACTGCAAGGCACCGTGACGGCTCAAGGACTAGCGATTC	
Cr-2276-F2	GAATCGCTAGTCCTTGAGCCGTCACGGTGCCTTGCAGTTCTTCTAAACGC	Amplification of the sequence downstream of the deletion region 2276-2284
Cr-2276-R2	GCAACGTTGTTGCCATTGCGCGATCGCTGTATTGCAGCATTTAGCG	
Cr-2384-F1	TGGCAGAAATTCGATATCTACAACTCGCAGATGCCAGTAATGACC	Amplification of the sequence upstream of the deletion region 2384-2392
Cr-2384-R1	GACTCTTTACCGTTGTGGGATACTCCGAACAGAGCAACGCACAGCCTGTC	
Cr-2384-F2	GACAGGCTGTGCGTTGCTCTGTTCGGAGTATCCCACAACGGTAAAGAGTC	Amplification of the sequence downstream of the deletion region 2384-2392
Cr-2384-R2	GCAACGTTGTTGCCATTGCGCCAACAACGGGCTTCAGTTCTCGATC	
Cr-2476-F1	TGGCAGAAATTCGATATCTAGGCACATCATCTCCATGCCTACTTAG	Amplification of the sequence upstream of the deletion region 2476-2490
Cr-2476-R1	CTATGTCAGTGGCGATCGCCTGACGCAAACTCCTACCGAAACAGAGACC	
Cr-2476-F2	GGTCTCTGTTTCGGTAGGAGTTTGCGTCAGGCGATCGCCACTGACATAG	Amplification of the sequence downstream of the deletion region 2476-2490
Cr-2476-R2	GCAACGTTGTTGCCATTGCGGACACCTTATAACCGCCACCGCC	
Cr-2573-F1	TGGCAGAAATTCGATATCTACGGAGTCGAAGAATCCCATAGCG	Amplification of the sequence upstream of the deletion region 2573-2578
Cr-2573-R1	GCAGGACTTTCAGGCTCCCATTGGGATATAACTCGGTACGAGGCAGC	
Cr-2573-F2	GCTGCCTCGTACCGAGTTATATCCCAATGGGAGCCTGAAAGTCCTGC	Amplification of the sequence downstream of the deletion region 2573-2578
Cr-2573-R2	GCAACGTTGTTGCCATTGCGGGATCAGGCTGCAATTAAAGCAGCC	
AarI-F	CACCAGTAGCAGATGTAAATGGCAATTTC	PCR verification of the editing plasmids
AarI-R	CGAATTTCTGCCATCGACGGTACCAAC	
BB-F	TGTAGATGCTACTATTCCTGTGCCTT	
BB-R	TTGCCATCACGACTGTGCTGGTCA	
Primers used for the PCR verification of single mutants generated using CRISPR/Cpf1b-Km		
Cr-0051-F′	GCATTAAGCCACTCTGTTTCCATACACC	PCR verification of *Δ0051-0062*
Cr-0051-R′	CGGAGCTAGTGGATGGCATTCCCC	
Cr-0051-F	GCCAGTAATTCCTCAACAGATCTGC	
Cr-0051-R	CCTAGTTTGAGAGGCATCCCCGA	
Cr-0233-F′	GCGAGCTTCAGTGCTGTATTCCGTC	
Cr-0233-R′	GGTCAGCAACTCAGACAGTGGAGC	

Cr-0233-F	GGATAGCGCAGCTCGTCGTCC	PCR verification of *Δ0233-0253*
Cr-0233-R	GGGCGGTCTGCGGGAAGAAATC	
Cr-0363-F′	CTATGGCCTGCGGGAATGGCTG	PCR verification of *Δ0363-0373*
Cr-0363-R′	CTGACTTTGGCTAGGCTCCAACTC	
Cr-0363-F	CGCTTGCAACTCGCCCAGGAGC	
Cr-0363-R	GCCGACAATCTCAGAACCTGCC	
Cr-0726-F′	GTCGTCGAAGAGCTGCGTCACTC	PCR verification of *Δ0726-0755*
Cr-0726-R′	GGATAAATTCGAATCCACTGGACGGG	
Cr-0726-F	GAACGAGATCAGCTCGGTCACG	
Cr-0726-R	CCTTGCTCCAGCCGGAAGGTC	
Cr-0763-F′	CAGCTTGGTTGAGCCCAGGCTC	PCR verification of *Δ0763-0774*
Cr-0763-R′	GGAAGTCTCTGTGGTTTCTGGTGCC	
Cr-0763-F	GATATTTCTGAAGCCCAGTTGCGCC	
Cr-0763-R	GTGTGGATCAAGGCCCTTGGTTTG	
Cr-0837-F′	CTCTATAACGTTGATCCCTGCGGAC	PCR verification of *Δ0837-0847*
Cr-0837-R′	CTCACTGCAACTGACGCTGGCTTCC	
Cr-0837-F	GCTACTCAGCAAGTTCAACTCAGCGG	
Cr-0837-R	CGCCAACCTTGGTCGTTTACAGC	
0837-F-3	GCCCGTACAGCCGATGAAAAGTC	
0837-R-3	CTGATTCGCTGTCGGGTAATCTGC	
Cr-0854-F′	GCGTCAATGCGCCCTACGTTTGG	PCR verification of *Δ0854-0860*
Cr-0854-R′	CTGATGCCCTGACGGACCTGACG	
Cr-0854-F	CAGCTGGTCCGTGAAATTACGCTG	
Cr-0854-R	CCAACTTGTGTCGCGAGCTGAC	
Cr-1152-F′	GAACGATCGCGATGGGCAGATTG	PCR verification of *Δ1152-1167*
Cr-1152-R′	CACAGTCACCCCTATTCAGTTCTCAGAG	
Cr-1152-F	CTCTGGATCTACTCGCCCCCG	
Cr-1152-R	CCAACTGAGGGCATCAAGCTGC	
Cr-1294-F′	CCTGAGTGAACTGCTGCCCCTC	PCR verification of *Δ1294-1305*
Cr-1294-R′	CTCAGATCAGGATACGGTGCGTTC	
Cr-1294-F	GTTCAGCCCCCCAATCCAGAACA	
Cr-1294-R	CTGCGATCAACCCTGAAGCATCTC	
Cr-1314-F′	GCTTGACCTACGCTGAGGCGATG	PCR verification of *Δ1314-1324*
Cr-1314-R′	CTCATGGCGATCGGGAAGGATGTC	
Cr-1314-F	CGGCAGCTTCGTAGACCGCCAG	
Cr-1314-R	GATGTCGCCAAACAGAAAGCCTTCC	
Cr-1354-F′	CGATAAGCTGCGTCAAGGCACTTAG	PCR verification of *Δ1354-1358*
Cr-1354-R′	CCGCTGGAACAGCAGGTTCATGG	
Cr-1354-F	CAGCGGCATATTGAGATCGAGAAGAATG	
Cr-1354-R	CCCGATTTGGAGGAAGCCCTTAG	
Cr-1393-F′	GAGATTTACCCTTGTTGCCAGTGC	PCR verification of *Δ1393-1400*
Cr-1393-R′	GATAAGACTCGCTAGCCATGAGTGG	
Cr-1393-F	CAATGGCGACAAAGAGGGCTTCC	

Cr-1393-R	CTGGCCAGCTGTAACGAACTTTGGC	
Cr-1533-F′	GTCAACACGGGGAATGCACCTG	PCR verification of *Δ1533-1551*
Cr-1533-R′	GTATTACGACGCCGATGCCAATCTG	
Cr-1533-F	CTCAACAGGGTTTTCTCTGGGG	
Cr-1533-R	GATGTGGATTTGTCTAAGCGGACGCC	
Cr-1567-F′	GACATCTTTACGGTGATTCGCCAGC	PCR verification of *Δ1567-1576*
Cr-1567-R′	CGCCCTTGAGACAGTCTCAGC	
Cr-1567-F	CCGCGATCGCTTCGTAGTAGAC	
Cr-1567-R	CGGTAGAACGTGAGCGTTACGGC	
Cr-1640-F′	CCTTGAGATTTCCCTGAAGTTCACGCC	PCR verification of *Δ1640-1650*
Cr-1640-R′	GCTGCCAAACTTGTGCTGCTGCC	
Cr-1640-F	CAACTTGCTGCGCAGGAATTGGG	
Cr-1640-R	CACCAAAGACCTGTCCTGCCAC	
Cr-1810-F′	GCCTTTGGCATGCACTTCTGCG	PCR verification of *Δ1810-1822*
Cr-1810-R′	GCAGACCTTAGGCTTCGCCCATG	
Cr-1810-F	CACTAGACGAAAGCAGGCTGAAGA	
Cr-1810-R	CCCCAGCCGAGCAAAAATATCCAC	
Cr-1865-F′	CCCTACATTCCCACTCAAGAGTGGC	PCR verification of *Δ1865-1878*
Cr-1865-R′	CCCTAGCTTGCAATTTCCATCGC	
Cr-1865-F	CAGCGATCGAGACCACTGGTTTCC	
Cr-1865-R	CGAAGAATCGCTCTCGGCGACTG	
1865-R-2	GATCGGTAATGTCACCAGCTCCGG	
Cr-2094-F′	GACACGCCCGATAACCTCAAGCAG	PCR verification of *Δ2094-2109*
Cr-2094-R′	CGATTGCCCTAGCCCAAGTTGC	
Cr-2094-F	CAGCGCGCGATCCTCAATCAG	
Cr-2094-R	GTGGTGACAAAGTTGCGGCCAC	
Cr-2169-F′	CTCTCGTTCGCTGGAATGTTCCC	PCR verification of *Δ2169-2187*
Cr-2169-R′	GCTTACAGGCCGAAGGTGCAACTC	
Cr-2169-F	GGCAACCAAGCCACAATCAGCA	
Cr-2169-R	CAGCTTCCGTGATTCCTACAGCG	
Cr-2276-F′	GGAAGAAGGCGAGAAGCCAGATC	PCR verification of *Δ2276-2284*
Cr-2276-R′	CATTTCCGAACTTTCAGTGGATTGCAGG	
Cr-2276-F	GGTGATTGCCATTCGGCATCTGC	
Cr-2276-R	CGGAGCATGCCTTGCTCCGTAG	
Cr-2384-F′	GCCTGCAATCAGCAAGGTGATCGATAC	PCR verification of *Δ2384-2392*
Cr-2384-R′	GTCTGTATCTTCGATGCGCAGGATG	
Cr-2384-F	CGCTCTCGACAACATGGCCAAC	
Cr-2384-R	CAGGTCGCTTGACCCGGATGTTC	
Cr-2476-F′	GATCGCGCAATGACCAAGTGGGTAC	PCR verification of *Δ2476-2490*
Cr-2476-R′	GTTCAAAGCGCTGTTGGTGGGTTTTG	
Cr-2476-F	CGACAGAGGTGCCCTGAGTTCG	
Cr-2476-R	CGGTTGGCATCACCTCAGTCAGC	
Cr-2573-F′	CCGTAGTCGCTGCCTAATTGAATCAG	

Cr-2573-R′	CTACTATGCCCTCCTGGGGATCC	PCR verification of *Δ2573-2578*
Cr-2573-F	CCTATTTCGCCGAGCCTCTCTCTG	
Cr-2573-R	CGTAAGGCTAAATACTCCTGTGTGACCG	
Primers used for the PCR verification of single mutants generated using CRISPR/Cpf1b-Sp		
0051-0062-F′	GCATTAAGCCACTCTGTTTCCATACACC	PCR verification of *Δ0051-0062*
0051-0062-R′	CGGAGCTAGTGGATGGCATTCCCC	
0051-0062-2	ACATTGCCTATCTCTCGGGTGATT	
0233-0253-F′	CTCTCGCCAAGAAGTTTTTGGGCA	PCR verification of *Δ0233-0253*
0233-0253-R′	TGGGAACTCTGGAAACAGGCCT	
0233-0253-2	ATCGACTAACTCCATCTCTCCTAGG	
0726-0755-F′	ACTACCAGCAGCAAGTCGTCGAA	PCR verification of *Δ0726-0755*
0726-0755-R′	GGTTCCTATGTGCCTGACTACATCT	
0726-0755-2	GTCTAGCTCGATCAGCTCGCCTC	
0763-0774-F′	CTACTGGTACTGGACCATGTTGAAG	PCR verification of *Δ0763-0774*
0763-0774-R′	AAGTCTCTGTGGTTTCTGGTGCCA	
0763-0774-2	CCTGCGTTTTCCGTTTCTCTTCTTGC	
0837-0847-F′	CTCTATAACGTTGATCCCTGCGGAC	PCR verification of *Δ0837-0847*
0837-0847-R′	CTCACTGCAACTGACGCTGGCTTCC	
0837-0847-2	GAGCCAGTTCTATCGCCACTATCCC	
1152-1167-F′	CAGATAGCGATTGCGCGAGCTGAG	PCR verification of *Δ1152-1167*
1152-1167-R′	GCGGTGCGCTTTGGAATACAGAATTC	
1152-1167-2	CTTTTGGGGCTAATCCCCTCAACAC	
1294-1305-F′	CCTGAGTGAACTGCTGCCCCTC	PCR verification of *Δ1294-1305*
1294-1305-R′	CTCAGATCAGGATACGGTGCGTTC	
1294-1305-2	TCGCCATCTTTAGCTGCACCG	
1314-1324-F′	GCTTGACCTACGCTGAGGCGATG	PCR verification of *Δ1314-1324*
1314-1324-R′	CTCATGGCGATCGGGAAGGATGTC	
1314-1324-2	CAGCCTAAAGCCCAAGGTTACGTC	
1354-1358-F′	CGATAAGCTGCGTCAAGGCACTTAG	PCR verification of *Δ1354-1358*
1354-1358-R′	CCGCTGGAACAGCAGGTTCATGG	
1354-1358-2	CCGCTGCGATCGCTAGACATAGCAG	
1533-1551-F′	GATGCCGCCGTTTGTATTGGTTG	PCR verification of *Δ1533-1551*
1533-1551-R′	GATGCCGCCGTTTGTATTGGTTG	
1533-1551-2	CTGTACTTCCTCTTCTGTGGCGCC	
1567-1576-F′	GACATCTTTACGGTGATTCGCCAGC	PCR verification of *Δ1567-1576*
1567-1576-R′	GACATCTTTACGGTGATTCGCCAGC	
1567-1576-2	CTCTTCTCCCCTCAAAGGCAGCG	
1810-1822-F′	CCCGCTAGCCAAATCCTCTTCACC	PCR verification of *Δ1810-1822*
1810-1822-R′	CGACTGTTGCTGATTTCATGACCCG	
1810-1822-2	CACGGCCTTGTTCAACTTGGCAG	
1865-1878-F′	GGGTTAGAAGCGCTACGCCATTTAG	PCR verification of *Δ1865-1878*
1865-1878-R′	CCCTAGCTTGCAATTTCCATCGCTG	
1865-1878-2	CACCGGCTTTGTAGGCGATGATC	

2094-2109-F′	CTCTACATCGCTGAGCAGGTGTTTC	PCR verification of *Δ2094-2109*
2094-2109-R′	GGCGATCGTGGCATGTCTGTTTCAC	
2094-2109-2	CTTCAACGGCTGTGAGTAGCGTCTG	
2169-2187-F′	GGTGGCTTGCGGTTCTATGATCG	PCR verification of *Δ2169-2187*
2169-2187-R′	ACAGGCCGAAGGTGCAACTCTAG	
2169-2187-2	GCCCAGGCTAAATCGACTGTTGCG	
2276-2284-F′	GATTCGCGGCCTATCGATGCAACTG	PCR verification of *Δ2276-2284*
2276-2284-R′	GTGAAACCACGTCTTTAACAACGGC	
2276-2284-2	CGATCATCCTGAGTTGGCAGAGGTC	
2384-2392-F′	GGCCGGATTGAAGCCCTGATCG	PCR verification of *Δ2384-2392*
2384-2392-R′	CGCAAACAACCAGTTGAAGACGGC	
2384-2392-2	GATCGCGGCAAAATCTTGCGCCTC	
Primers used for the PCR verification of multiple deletion strains		
Cr-0051-F′	GCATTAAGCCACTCTGTTTCCATACACC	PCR verification of the 0051-0062 deletion in the double, quadruple, quintuple, sextuple, septuple deletion strain.
Cr-0051-R′	CGGAGCTAGTGGATGGCATTCCCC	
Cr-0051-F	GCCAGTAATTCCTCAACAGATCTGC	PCR verification of the 0051-0062 deletion in the double, sextuple, septuple deletion strain.
Cr-0051-R	CCTAGTTTGAGAGGCATCCCCGA	
Cr-0051-F	GCCAGTAATTCCTCAACAGATCTGC	PCR verification of the 0051-0062 deletion in the quadruple, quintuple deletion strain.
Cr0051-R-1	CTGCTAAAACCTCAAGGCCGGGC	
Cr-0726-F′	GTCGTCGAAGAGCTGCGTCACTC	PCR verification of the 0726-0755 deletion in the septuple deletion strain.
Cr-0726-R′	GGATAAATTCGAATCCACTGGACGGG	
Cr-0726-F-1	TGACAGCCTGACCAAAGCACTCG	
Cr-0726-R-1	CCTCGAACTGCGTCTTTTGGATCGT	
Cr-0837-F′	CTCTATAACGTTGATCCCTGCGGAC	PCR verification of the 0837-0847 deletion in the quintuple, sextuple, septuple deletion strain.
Cr-0837-R′	CTCACTGCAACTGACGCTGGCTTCC	
Cr-0837-F-1	TTCCTTGCGGTGCATCAGGATCAG	PCR verification of the 0837-0847 deletion in the quintuple deletion strain.
Cr-0837-R-1	GTTTACACCAGCAGCCACCGTTCT	
Cr-0837-F-3	GCCCGTACAGCCGATGAAAAGTC	PCR verification of the 0837-0847 deletion in the sextuple, septuple deletion strain.
Cr-0837-R-3	CTGATTCGCTGTCGGGTAATCTGC	
Cr-1354-F′	CGATAAGCTGCGTCAAGGCACTTAG	PCR verification of the 1354-1358 deletion in the quadruple, quintuple, sextuple, septuple deletion strain.
Cr-1354-R′	CCGCTGGAACAGCAGGTTCATGG	
Cr-1354-F	CAGCGGCATATTGAGATCGAGAAGAATG	
Cr-1354-R-1	CGCCAGGTTTTCCAGAATCTGTTG	
Cr-1393-F′-1	CGAAATGCAAAGTGCTGACCTGCG	PCR verification of the 1393-1400 deletion in the sextuple deletion strain.
Cr-1393-R’-1	CGAGCTAGTTTGGCAGGATGCAGAC	
Cr-1393-F-1	CGGTACAAGTGGCAGCACATCTTC	

Cr-1393-R-1	GGCAACAAGATGCTCTCACAGCCC	
Cr-1810-F′	GCCTTTGGCATGCACTTCTGCG	PCR verification of the 1810-1822 deletion in the sextuple, septuple deletion strain.
Cr-1810-R′	GCAGACCTTAGGCTTCGCCCATG	
Cr-1810-F-1	CAGTAGGACGCATCCTCGGATCAC	
Cr-1810-R-1	CTGCCACTCTTGCGATGATGGATG	
Cr-1865-F′	CCCTACATTCCCACTCAAGAGTGGC	PCR verification of the 1865-1878 deletion in the double, quadruple, quintuple, sextuple, septuple deletion strain.
Cr-1865-R′	CCCTAGCTTGCAATTTCCATCGC	
Cr-1865-F	CAGCGATCGAGACCACTGGTTTCC	PCR verification of the 1865-1878 deletion in the double deletion strain.
Cr-1865-R	CGAAGAATCGCTCTCGGCGACTG	
Cr-1865-F-1	GTGCGTGCAATATTGCCGGTATTG	PCR verification of the 1865-1878 deletion in the quadruple, quintuple deletion strain.
Cr-1865-R-1	GAGATGTTCACCTTCTCGGCG	
Cr-1865-F	CAGCGATCGAGACCACTGGTTTCC	PCR verification of the 1865-1878 in the sextuple, septuple deletion strain.
Cr-1865-R-2	GATCGGTAATGTCACCAGCTCCGG	
Cr-2276-F′	GGAAGAAGGCGAGAAGCCAGATC	PCR verification of the 2276-2284 in the sextuple, septuple deletion strain.
Cr-2276-R′	CATTTCCGAACTTTCAGTGGATTGCAGG	
Cr-2276-F′-1	GTGAGGATAAGCAACCGTTAGCTGG	PCR verification of the 2276-2284 deletion in the quadruple, quintuple deletion strain.
Cr-2276-R′-1	GCAGTCATGCTCGGGAAAGCCACG	
Cr-2276-F	GGTGATTGCCATTCGGCATCTGC	PCR verification of the 2276-2284 deletion in the quadruple, quintuple, sextuple, septuple deletion strain.
Cr-2276-R	CGGAGCATGCCTTGCTCCGTAG	
0051-0062-F′	GCATTAAGCCACTCTGTTTCCATACACC	PCR verification of the 0051-0062 deletion in the triple deletion strain.
0051-0062-R′	CGGAGCTAGTGGATGGCATTCCCC	
0051-0062-2	ACATTGCCTATCTCTCGGGTGATT	
1865-1878-F′	GGGTTAGAAGCGCTACGCCATTTAG	PCR verification of the 1865-1878 deletion in the triple deletion strain.
1865-1878-R′	CCCTAGCTTGCAATTTCCATCGCTG	
1865-1878-2	CACCGGCTTTGTAGGCGATGATC	
2276-2284-F′	GATTCGCGGCCTATCGATGCAACTG	PCR verification of the 2276-2284 deletion in the triple deletion strain.
2276-2284-R′	GTGAAACCACGTCTTTAACAACGGC	
2276-2284-2	CGATCATCCTGAGTTGGCAGAGGTC	

Table S3. List of 69 editing plasmids in this study

Plasmid	Resistance	Descriptions
pHB6860	Sp	The plasmid with the gRNA-0051 sequence and homologous arms for deleting the genomic region 0051-0062
pHB6861	Sp	The plasmid with the gRNA-0233 sequence and homologous arms for deleting the genomic region 0233-0253
pHB6906	Sp	The plasmid with the gRNA-0363 sequence and homologous arms for deleting the genomic region 0363-0373

pHB6862	Sp	The plasmid with the gRNA-0726 sequence and homologous arms for deleting the genomic region 0726-0755
pHB6863	Sp	The plasmid with the gRNA-0763 sequence and homologous arms for deleting the genomic region 0763-0774
pHB6864	Sp	The plasmid with the gRNA-0837 sequence and homologous arms for deleting the genomic region 0837-0847
pHB6865	Sp	The plasmid with the gRNA-0854 sequence and homologous arms for deleting the genomic region 0854-0860
pHB6866	Sp	The plasmid with the gRNA-1152 sequence and homologous arms for deleting the genomic region 1152-1167
pHB6867	Sp	The plasmid with the gRNA-1294 sequence and homologous arms for deleting the genomic region 1294-1305
pHB6868	Sp	The plasmid with the gRNA-1314 sequence and homologous arms for deleting the genomic region 1314-1324
pHB6869	Sp	The plasmid with the gRNA-1354 sequence and homologous arms for deleting the genomic region 1354-1358
pHB6870	Sp	The plasmid with the gRNA-1393 sequence and homologous arms for deleting the genomic region 1393-1400
pHB6907	Sp	The plasmid with the gRNA-1533 sequence and homologous arms for deleting the genomic region 1533-1551
pHB6871	Sp	The plasmid with the gRNA-1567 sequence and homologous arms for deleting the genomic region 1567-1576
pHB6872	Sp	The plasmid with the gRNA-1640 sequence for deleting the genomic region 1640-1650
pHB6873	Sp	The plasmid with the gRNA-1810 sequence and homologous arms for deleting the genomic region 1810-1822
pHB6910	Sp	The plasmid with the gRNA-1865 sequence and homologous arms for deleting the genomic region 1865-1878
pHB6874	Sp	The plasmid with the gRNA-2094 sequence and homologous arms for deleting the genomic region 2094-2109
pHB6911	Sp	The plasmid with the gRNA-2169 sequence and homologous arms for deleting the genomic region 2169-2187
pHB6912	Sp	The plasmid with the gRNA-2276 sequence and homologous arms for deleting the genomic region 2276-2284
pHB6875	Sp	The plasmid with the gRNA-2384 sequence and homologous arms for deleting the genomic region 2384-2392
pHB6876	Sp	The plasmid with the gRNA-2476 sequence and homologous arms for deleting the genomic region 2476-2490
pHB6877	Sp	The plasmid with the gRNA-2573 sequence and homologous arms for deleting the genomic region 2573-2578
pHB6588	Km	The plasmid with the gRNA-0051-1 sequence and homologous arms for deleting the genomic region 0051-0062
pHB6589	Km	The plasmid with the gRNA-0051-2 sequence and homologous arms for deleting the genomic region 0051-0062

pHB6848	Km	The plasmid with the gRNA-0233-1 sequence and homologous arms for deleting the genomic region 0233-0253
pHB6818	Km	The plasmid with the gRNA-0233-2 sequence and homologous arms for deleting the genomic region 0233-0253
pHB6856	Km	The plasmid with the gRNA-0363-1 sequence and homologous arms for deleting the genomic region 0363-0373
pHB6857	Km	The plasmid with the gRNA-0363-2 sequence and homologous arms for deleting the genomic region 0363-0373
pHB6881	Km	The plasmid with the gRNA-0763-1 sequence and homologous arms for deleting the genomic region 0763-0774
pHB6849	Km	The plasmid with the gRNA-0763-2 sequence and homologous arms for deleting the genomic region 0763-0774
pHB6878	Km	The plasmid with the gRNA-0837-1 sequence and homologous arms for deleting the genomic region 0837-0847
pHB6879	Km	The plasmid with the gRNA-0837-2 sequence and homologous arms for deleting the genomic region 0837-0847
pHB6880	Km	The plasmid with the gRNA-0854-1 sequence and homologous arms for deleting the genomic region 0854-0860
pHB6817	Km	The plasmid with the gRNA-0854-2 sequence and homologous arms for deleting the genomic region 0854-0860
pHB6850	Km	The plasmid with the gRNA-2476-1 sequence and homologous arms for deleting the genomic region 2476-2490
pHB6819	Km	The plasmid with the gRNA-2476-2 sequence and homologous arms for deleting the genomic region 2476-2490
pHB6729	Km	The plasmid with the gRNA-0726-1 sequence and homologous arms for deleting the genomic region 0726-0755
pHB6730	Km	The plasmid with the gRNA-0726-2 sequence and homologous arms for deleting the genomic region 0726-0755
pHB6731	Km	The plasmid with the gRNA-1152-1 sequence and homologous arms for deleting the genomic region 1152-1167
pHB6732	Km	The plasmid with the gRNA-1152-2 sequence and homologous arms for deleting the genomic region 1152-1167
pHB6884	Km	The plasmid with the gRNA-1294-1 sequence and homologous arms for deleting the genomic region 1294-1305
pHB6885	Km	The plasmid with the gRNA-1294-2 sequence and homologous arms for deleting the genomic region 1294-1305
pHB6886	Km	The plasmid with the gRNA-1314-1 sequence and homologous arms for deleting the genomic region 1314-1324
pHB6887	Km	The plasmid with the gRNA-1314-2 sequence and homologous arms for deleting the genomic region 1314-1324
pHB6898	Km	The plasmid with the gRNA-1810-1 sequence and homologous arms for deleting the genomic region 1810-1822
pHB6899	Km	The plasmid with the gRNA-1810-2 sequence and homologous arms for deleting the genomic region 1810-1822

pHB6883	Km	The plasmid with the gRNA-2276-1 sequence and homologous arms for deleting the genomic region 2276-2284
pHB6822	Km	The plasmid with the gRNA-2276-2 sequence and homologous arms for deleting the genomic region 2276-2284
pHB6888	Km	The plasmid with the gRNA-2573-1 sequence and homologous arms for deleting the genomic region 2573-2578
pHB6889	Km	The plasmid with the gRNA-2573-2 sequence and homologous arms for deleting the genomic region 2573-2578
pHB6858	Km	The plasmid with the gRNA-1354-1 sequence and homologous arms for deleting the genomic region 1354-1358
pHB6859	Km	The plasmid with the gRNA-1354-2 sequence and homologous arms for deleting the genomic region 1354-1358
pHB6900	Km	The plasmid with the gRNA-1865-1 sequence and homologous arms for deleting the genomic region 1865-1878
pHB6901	Km	The plasmid with the gRNA-1865-2 sequence and homologous arms for deleting the genomic region 1865-1878
pHB6904	Km	The plasmid with the gRNA-2384-1 sequence and homologous arms for deleting the genomic region 2384-2392
pHB6905	Km	The plasmid with the gRNA-2384-2 sequence and homologous arms for deleting the genomic region 2384-2392
pHB6846	Km	The plasmid with the gRNA-2094-1 sequence and homologous arms for deleting the genomic region 2094-2109
pHB6882	Km	The plasmid with the gRNA-2094-2 sequence and homologous arms for deleting the genomic region 2094-2109
pHB6902	Km	The plasmid with the gRNA-2169-1 sequence and homologous arms for deleting the genomic region 2169-2187
pHB6903	Km	The plasmid with the gRNA-2169-2 sequence and homologous arms for deleting the genomic region 2169-2187
pHB6892	Km	The plasmid with the gRNA-1533-1 sequence and homologous arms for deleting the genomic region 1533-1551
pHB6893	Km	The plasmid with the gRNA-1533-2 sequence and homologous arms for deleting the genomic region 1533-1551
pHB6894	Km	The plasmid with the gRNA-1567-1 sequence and homologous arms for deleting the genomic region 1567-1576
pHB6895	Km	The plasmid with the gRNA-1567-2 sequence and homologous arms for deleting the genomic region 1567-1576
pHB6890	Km	The plasmid with the gRNA-1393-1 sequence and homologous arms for deleting the genomic region 1393-1400
pHB6891	Km	The plasmid with the gRNA-1393-2 sequenc and homologous arms e for deleting the genomic region 1393-1400
pHB6896	Km	The plasmid with the gRNA-1640-1 sequence and homologous arms for deleting the genomic region 1640-1650
pHB6897	Km	The plasmid with the gRNA-1640-2 sequence and homologous arms for deleting the genomic region 1640-1650

Table S4. List of nonessential-gene regions over 10 kb

No.	The nonessential genomic regions	Lengths（bp）
1	*Synechococcus* 7942_0051-0062	10637
2	*Synechococcus* 7942 _0233-0253	19000
3	*Synechococcus* 7942 _0363-0373	12515
4	*Synechococcus* 7942 _0726-0755	29942
5	*Synechococcus* 7942_0763-0774	10114
6	*Synechococcus* 7942_0837-0847	10054
7	*Synechococcus* 7942_0854-0860	13713
8	*Synechococcus* 7942_1152-1167	23660
9	*Synechococcus* 7942_1294-1305	14710
10	*Synechococcus* 7942_1314-1324	13054
11	*Synechococcus* 7942_1354-1358	11292
12	*Synechococcus* 7942_1393-1400	9873
13	*Synechococcus* 7942_1533-1551	16472
14	*Synechococcus* 7942_1567-1576	14314
15	*Synechococcus* 7942_1640-1650	12792
16	*Synechococcus* 7942_1810-1822	15548
17	*Synechococcus* 7942_1865-1878	16181
18	*Synechococcus* 7942_2094-2109	14707
19	*Synechococcus* 7942_2169-2189	14942
20	*Synechococcus* 7942_2276-2284	10891
21	*Synechococcus* 7942_2384-2392	16121
22	*Synechococcus* 7942_2476-2490	12628
23	*Synechococcus* 7942_2573-2578	11050
